# The multivalency game ruling the biology of immunity

**DOI:** 10.1063/5.0166165

**Published:** 2023-12-29

**Authors:** Lara Victoria Aiassa, Giuseppe Battaglia, Loris Rizzello

**Affiliations:** 1 Institute for Bioengineering of Catalunya (IBEC), Barcelona Institute of Science and Technology (BIST), Barcelona, Spain; 2 Department of Biomedical Sciences, University of Barcelona, Barcelona, Spain; 3 Catalan Institution for Research and Advanced Studies (ICREA), Barcelona, Spain; 4 Department of Pharmaceutical Sciences, (DISFARM) University of Milan, Milan, Italy; 5 National Institute of Molecular Genetics (INGM), Milan, Italy

## Abstract

Macrophages play a crucial role in our immune system, preserving tissue health and defending against harmful pathogens. This article examines the diversity of macrophages influenced by tissue-specific functions and developmental origins, both in normal and disease conditions. Understanding the spectrum of macrophage activation states, especially in pathological situations where they contribute significantly to disease progression, is essential to develop targeted therapies effectively. These states are characterized by unique receptor compositions and phenotypes, but they share commonalities. Traditional drugs that target individual entities are often insufficient. A promising approach involves using multivalent systems adorned with multiple ligands to selectively target specific macrophage populations based on their phenotype. Achieving this requires constructing supramolecular structures, typically at the nanoscale. This review explores the theoretical foundation of engineered multivalent nanosystems, dissecting the key parameters governing specific interactions. The goal is to design targeting systems based on distinct cell phenotypes, providing a pragmatic approach to navigating macrophage heterogeneity's complexities for more effective therapeutic interventions.

## I. INTRODUCTION

Macrophages are cells that are part of the innate immune system with the function to defend the organism from external insult including physical injury or/and exposure to toxins or infections. They are present throughout the body and have a crucial role in maintaining tissue homeostasis and performing essential tissue-specific functions.^1^ The first response to any insult is the so-called inflammatory process, mediated by resident macrophages that activate signaling molecules known as cytokines to recruit other macrophages and immune cells to the site of injury/infection.

The resident macrophages are hosted and instructed via local environmental stimuli to perform tissue-specialized functions within the different organs, ultimately shaping macrophage identity.^2^ The functional and organotrophic specialization of macrophages leads to high heterogeneity. Macrophages can also change their state, switching from a basal state, where they perform tissue-specific homeostatic functions, to an inflammatory condition, where they initiate an immune response to eliminate invading pathogens. Moreover, they can adopt an anti-inflammatory state, where they promote tissue repair. Such a process, known as plasticity, is a defining feature of macrophage.^3,4^ In response to environmental stimuli, macrophages can change their effector function, which results in changes in receptor expression, cytokine, and chemokine production, giving place to different cell phenotypes.^5,6^ Macrophage plasticity provides an extra dimension to macrophage heterogeneity upon homeostasis disruption where disease-associated environmental stimuli further shape macrophage phenotype.^7,8^ The study of macrophage heterogeneity is particularly important as they lay between tissue homeostasis and pathogenesis in virtually all human tissues. A better understanding of macrophage function, especially in disease processes, and a better characterization of the diversity of phenotypes that they can adopt is fundamental for developing novel macrophage-targeted therapies.

This review explores the types of resident macrophages found in different tissues and their specific functions in the steady state. Recent research has emphasized how their origin and developmental pathway, known as macrophage ontogeny, can affect their characteristics.^9–12^ We then examine the amplified heterogeneity of macrophage activation states during pathological conditions. Using tuberculosis (TB) as an example, we highlight recent findings on how cell lineage, tissue environment, and bacterial pathogenic factors affect the macrophage response. TB is a highly infectious disease caused by *Mycobacterium tuberculosis* (*Mtb*), which invades the body through the airways.^13^ *Mtb* subverts host immunity to establish its survival within the macrophage niche, ultimately escaping and spreading the infection to healthy individuals.^14^ Studies show that different macrophage lineages respond differently to *Mtb*, resulting in restrictive or permissive phenotypes influencing the onset and spread of infection.^15,16^ The dynamism of the macrophage activation state plays a central role in driving disease progression. As a result, an enhanced comprehension of macrophage diversity is crucial to develop therapeutic strategies targeted at macrophages to combat intracellular pathogens like *Mtb*.

In the second part of the review, we will discuss the theoretical framework underpinning multivalent interactions. Multivalency refers to the interaction of multiple ligands attached to a single entity, with numerous receptors on another entity, all happening simultaneously.^17^ Multivalency is a crucial principle in nature, enabling the formation of robust yet reversible interactions vital for signal transduction, recognition, and adhesion processes.^18^ An illustrative instance is seen in the recognition of pathogens by macrophages, where multiple receptors must engage to identify an exogenous particle and trigger a response. Conversely, viral particles employ various copies of a protein to interact with entry receptors on specific cells, initiating infection.^19^ In both scenarios, the simultaneous binding of numerous ligand–receptor pairs reinforces the interaction, while the combinatorial effects enhance binding selectivity.

Similarly, multivalent systems can be engineered to target a specific cell population that can be exploited for therapeutic purpose. Nanoscopic scaffolds can be decorated with ligands to enhance weak individual interactions through collective binding, limiting association to a specific cell population expressing a unique receptor profile, a phenomenon that we address as “phenotypic targeting.” We dive into the theoretical principles of such a multicomponent targeting using statistical mechanics first principles. The rules governing the interaction of the multivalent scaffold are determined by the ligand–receptor interaction, which faces opposition from two sources of steric potential: the polymer brush coating the multivalent scaffold and the often-overlooked cell glycocalyx, which is a sugar-rich cushion covering the cell membrane that can impede the interaction. Developing novel therapeutic approaches that selectively target surfaces displaying a unique receptor density represents a promising strategy in nanomedicine for developing anti-inflammatory, anti-tumor, and bactericidal therapies.

The review aims to combine biology and physics disciplines from a comprehensive approach to both fields. First, addressing the complexity of macrophage function and its evident phenotypic heterogeneity that becomes essential for the development of novel targeted therapeutic systems. Second, assessing the nanoparticle design principles to decouple the relevant parameters governing selective interactions for the design of superselective targeting systems for disease-associated cells.

## II. MACROPHAGE BIOLOGY

Macrophages are professional phagocytic cells specialized in pathogen recognition and immune response but are also involved in tissue remodeling and homeostasis and coordinate tissue repair.^20^ Early during development, macrophages infiltrate and colonize every organ in the body, developing distinct functional specificity based on the tissue microenvironment.^21^

Macrophages are broadly classified into two subtypes: tissue-resident macrophages (TRMs) and infiltrating macrophages, also known as monocyte-derived macrophages (MDMs).^22^ TRMs are present ubiquitously in every tissue displaying great anatomical and functional diversity.^20,21,23^ Examples of TRMs are liver Kupffer cells, microglia in the central nervous system (CNS), alveolar macrophages (AMs) in the lungs, and splenic red pulp macrophages, which perform homeostatic functions in their respective tissues in the steady state.^24–27^ Under inflammatory conditions, TRMs are complemented by recruited monocytes that differentiate *in situ* into MDMs. It is important to distinguish resident macrophages that were present before the insult from macrophages that developed during the inflammation. TRMs and MDMs differ in developmental lineages with evidence of non-redundant functions during steady state and disease.^28^

For over four decades, the prevailing view of macrophage origin sustained that macrophages derive exclusively from circulating blood monocytes, the latest being derived from “pro-monocytes” from the bone marrow (BM).^29^ However, the paradigm has changed, and it is now established and well-documented that most TRMs arise from embryonic precursors that populate these tissues before birth and self-maintain throughout adulthood, independent from BM-derived precursors in the steady state.^1,22,23,30^ Several hypotheses attempt to explain the embryonic origin of TRMs reviewed in Refs. 20, 31, and 32. These models share the notion that yolk-sac (YS)-derived progenitor cells arise in two waves that differentially contribute to adult macrophages. An early wave of YS-derived primitive progenitor cells seeds the brain and other fetal tissues to give rise to all tissue macrophages. After the onset of the embryo blood circulation, a later wave of YS-derived definite progenitor cells, known as erythro-myeloid precursors (EMPs) that colonize and expand only in the fetal liver, differentiate into fetal liver monocytes (FL-MOs), which subsequently replace fetal macrophages in all tissues during embryogenesis, except for microglia. After postnatal formation of bone, FL hematopoiesis declines and is replaced by BM hematopoiesis during adulthood.^33–35^ BM progenitors are known as hematopoietic stem cells (HSCs) that derive from the aorta-gonad-mesonephros in the early embryo (pre-HSCs) that migrate to the FL where they expand and colonize the BM establishing the definite adult hematopoietic system.^21^

**FIG. 1.**
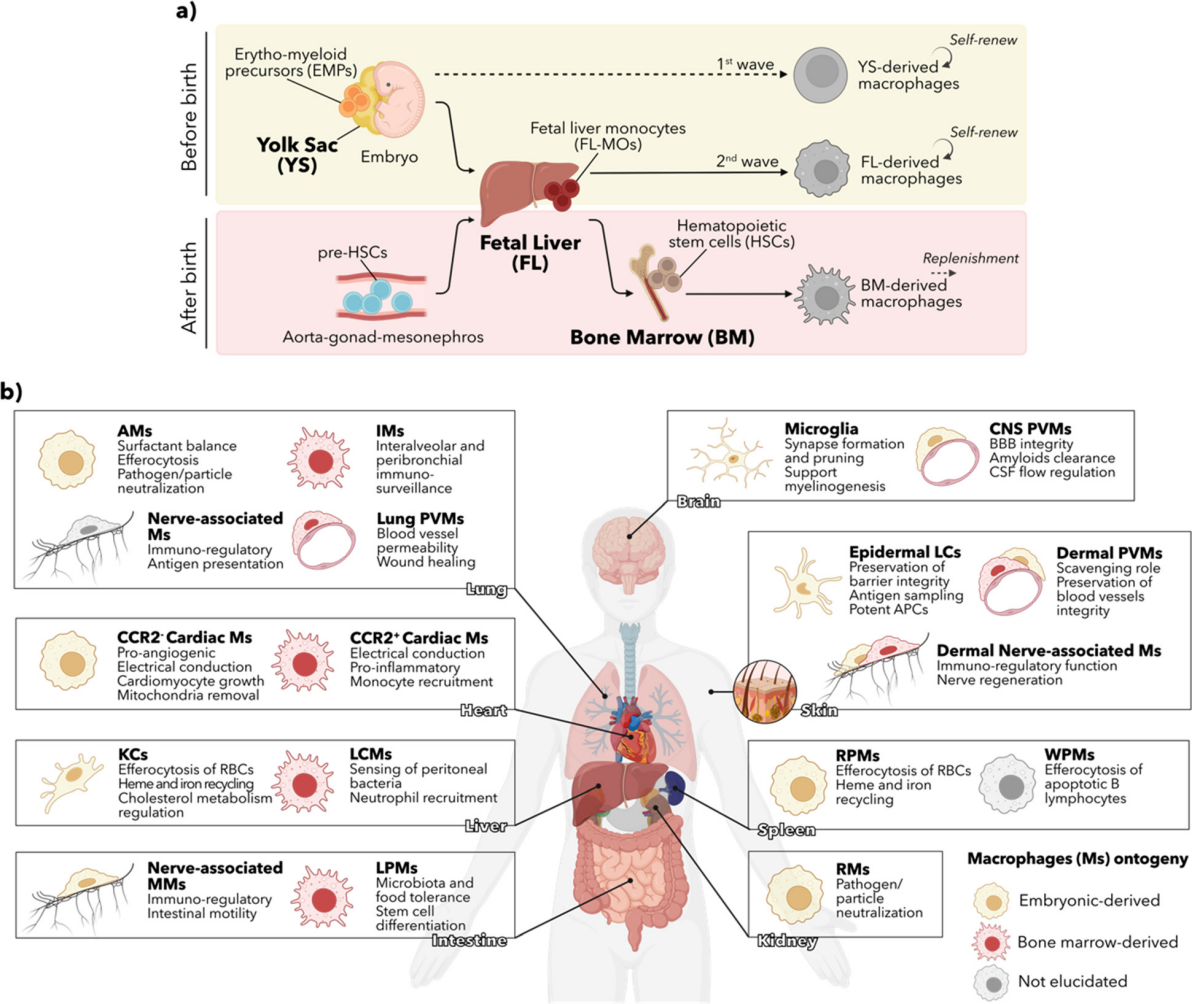
Ontogeny and functional specificity of tissue resident macrophages (TRMs). (a) During development, fetal tissues' TRMs are seeded trough two distinct waves of precursor cells. The initial wave consists of embryonic precursors from the yolk sac (YS), which give rise to all tissue macrophages including those found in the brain. A subsequent wave of YS-derived precursors colonizes and expands in the fetal liver (FL), differentiating into FL monocytes (FL-MOs) that replace most fetal macrophages, excluding microglia. FL-derived macrophages persist into adulthood through local proliferation. After birth, precursor hematopoietic stem cells (pre-HSCs) migrate and proliferate within the FL before colonizing the bone marrow (BM), where they establish the definitive adult hematopoietic system. BM-derived HSCs continuously generate specific subsets of TRMs, each organ with different kinetics. The three current supported sources of TRMs are the YS (exclusive to microglia), the FL, and the BM (only source sustained throughout adulthood). (b) Macrophages are a highly heterogeneous population of cells completing essential tissue-specific functions that promote tissue integrity. Despite their currently underappreciated regulatory role, macrophages actively counteract and limit changes in their local environment contributing to the preservation of tissue “balance” or homeostasis, as first pointed out by Elie Metchnikoff (Immunology Nobel, 1908). Best known by their immunological function, TRMs shared function includes their immune surveillance role, sampling every tissue and protecting the organism from external insults. Tissue macrophage populations have a mixed embryonic and postnatal bone marrow origin. AMs: alveolar macrophages, IMs: interstitial macrophages, PVMs: perivascular macrophages, CSF: cerebrospinal fluid, KCs: Kupffer cells, LCMs: liver capsular macrophages, MMs: muscularis macrophages, LPMs: *Lamina propria* macrophages, LCs: Langerhans cells, RPMs: red pulp macrophages, WPMs: white pulp macrophages, RMs: renal macrophages, RBCs: red blood cells.

Therefore, there are currently three supported sources of TRMs [Fig. 1(a)]. There are the YS-derived progenitors that populate all tissues in the initial stage of embryogenesis, but prevail in adulthood exclusively in the brain as self-sustained resident macrophages (microglia).^36^ A second embryonic source consists of FL MOs that colonize all the embryonic tissues after the YS colonization (except for the brain). They possess self-sustained capability in steady-state conditions and include AMs,^22,30,37^ Kupffer cells,^30,38^ kidney macrophages,^33^ most of the pancreatic macrophages,^34,39^ splenic red pulp and peritoneal macrophages,^30^ and the majority of cardiac (CCR2^−^) macrophages.^38^ The third linage, the BM-derived macrophages is postnatal and persists through adulthood maintaining a constant supply of TRMs after birth in the intestine,^40^ the dermis,^41^ and the heart (CCR2^+^ macrophages)^38^ in the steady-state condition with a marked difference in the kinetic replacement of these cells.^32^

Each organ comprises embryonic-derived and adult-derived macrophage subsets, with mixed populations like in the heart, dermis, pancreas, and liver as discussed before. Also, each organ dynamically modulates the degree to which circulating monocytes replace TRMs after birth. Although the embryonic ontogeny of macrophages is now accepted, the precise identification of the progenitors giving rise to fetal macrophages remains a main challenge, with the need to reconcile the data generated in different fate-mapping models. A new conceptual framework is emerging, which provides a better clue about macrophage origin and function, their evident symbiotic dependency, and their contribution to tissue preservation or disease progression. Consequently, distinct ontogeny has become a defining feature of macrophage biology, with ongoing investigations to identify to what extent it is determining macrophage functionality.

### A. TRMs functional specificity

Local environmental stimuli induce macrophage genetic and epigenetic alterations leading to long-term phenotypical changes that shape cellular identity driving tissue-specialized functions of different TRMs populations.^2^ Modulation of the TRMs-phenotype can be tissue-specific and derive from the cell's local environment, the so-called “tissue niche,” that provides signals for the maturation of functional TRMs in a time-dependent manner.^17^ Researchers used genetically modified mice lacking adult macrophages to illustrate this concept. They found that precursor cells from different sources (YS, FL, and BM) could successfully populate empty niches in neonate mice and develop mature tissue-resident macrophages (TRMs) in the lungs, which effectively prevented lung disorders.^42^ However, when adult macrophages were transferred to neonate mice lacking their own resident macrophages, these donor-derived cells had limited adaptability and could not efficiently prevent the lung disorder, indicating that adult macrophages are imprinted with specific functions and have limited flexibility due to their unique transcriptional programs.^2^

Stroma-driven conditioning of TRMs starts early during development. For example, peroxisome proliferator-activated receptor-γ (PPAR-γ) is the main transcription factor that regulates the perinatal development and signature gene expression of AMs.^43^ PPAR-γ induction is driven by the granulocyte-macrophage colony-stimulating factor (GM-CSF) production by the lung stroma,^43^ specifically alveolar epithelial type 2 cells,^44^ and has a crucial role in AM development shortly after birth.^37^ In addition, the effective modulation of macrophage identity may depend on the preservation of niche integrity. Previous studies have shown that resident alveolar macrophages (AMs) with impaired phagocytosis could regain their functionality after being transferred into naive mice. However, naive resident AMs were unable to function properly when transferred into the lungs of recently infected mice, highlighting the significant influence of the cellular environment in shaping macrophage function.^45^

Altogether, these findings reinforce the concept of tissue imprinting and stroma-derived signals as the dominant factor shaping TRMs functional specificity. This section briefly discusses TRMs' functions across different tissues in the steady state.

#### 1. Cardiac macrophages

In the adult heart (mice and humans), two main populations have been identified and distinguished by the expression of CCR2, a chemokine receptor important for migration.^12^ In healthy individuals, most cardiac macrophages lack CCR2 and are labeled as CCR2(–). They are embryonically (EB) derived and self-maintain throughout life.^12^ CCR2(+) macrophages are BM-derived, recruited early during development, and are maintained through monocyte recruitment in a CCR2-dependent manner, and subsequent proliferation.^12,38^ Shared functions include local cardiac sampling and engulfment of debris,^46^ more prominent in CCR2(–) macrophages,^47^ and facilitating electrical cardiac conduction via gap junctions with cardiomyocites.^48^

Specifically, CCR2(–) macrophages display an essential proangiogenic function mediating coronary blood vessel development.^49^ They also promote cardiomyocyte growth by insulin-like growth factor-1 (Igf1), especially relevant to withstand hypertensive stress.^50^ Furthermore, they actively promote correct cardiac function by removing defective mitochondria expelled by cardiomyocytes that otherwise accumulate extracellularly and induce inflammation. Conversely, CCR2(+) macrophage function is mainly reported in heart-associated diseases with a pro-inflammatory behavior and monocyte recruitment.^10^

#### 2. CNS macrophages

The main resident macrophages of the brain parenchyma are referred to as microglia.^51^ They are seeded at the initial stage of embryogenesis from YS-derived progenitor cells, and they perform a crucial role in brain development during the prenatal stage.^36^ Microglia play a role in guiding the formation of neuronal circuits during brain development.^52^ They are also involved in both the synapse formation and control their number by pruning them.^53,54^ In the adult brain, microglia support oligodendrocyte progenitor cells' survival and differentiation to drive myelinogenesis,^55^ i.e., the formation of sheaths that envelop axons.^56^

Brain TRMs are highly heterogeneous with various defined populations (by markers expression and different tissue localization) with overlapping and distinct functions.^51^ In addition to microglia, a second population of TRMs, called perivascular macrophages (PVMs), is located in close contact with brain vessels. PVMs are strategically located at the interface between blood and the brain parenchyma, supporting the blood–brain barrier's (BBB) integrity.^57^ As microglia, PVMs perform a phagocytic scavenging and immune surveillance function. They are also involved in clearing abnormal protein aggregates, such as amyloid plaques causative of neurodegenerative diseases such as Alzheimer's disease.^58,59^ Moreover, PVMs regulate the cerebrospinal fluid (CSF), remodeling the extracellular matrix (ECM). Accumulation of ECM proteins obstructs CSF access to perivascular spaces and impairs CNS perfusion and clearance with alterations in brain homeostasis.^60^

#### 3. Intestinal macrophages

The gut is the largest mucosal membrane in the body and remains in constant contact with both food and pathogenic antigens. Two main macrophage populations have been identified. *Lamina propria* macrophages (LPMs) are located underneath the epithelial layer near the gut lumen, sampling luminal pathogenic bacteria, and are essential for the establishing oral tolerance to food antigens.^61^ LPMs are constantly replenished during adulthood and express pro-inflammatory genes, such as IL-1β and IL-12,^62^ while expressing elevated levels of IL-10 involved in microbiota and dietary antigens tolerance.^63^ Importantly, they also modulate intestinal stem cell differentiation that gives origin to all the epithelial cell populations, being the intestinal epithelial layer one of the most rapidly self-renewing mucosal surface.^63^ A second population of macrophages, residing distal from the lumen in the submucosa and deeper in the *muscularis externa* was found named *Muscularis* macrophages (MMs). They derive from embryonic precursors and self-sustain in a steady state.^64^ MMs exhibit a tissue-protective phenotype, based on the expression of anti-inflammatory markers such as CD163, MRC1, and IL-10, and they closely interact with the enteric nervous system regulating intestinal motility.^62^

#### 4. Kidney macrophages

The kidneys regulate the body's water balance, blood pressure, acid–base levels, and electrolyte concentrations via plasma filtration at the nephron (the functional units of the kidneys) to maintain overall homeostasis.^65^ Renal macrophages (RMs) are embryonically derived^33,66^ in the steady state and participate in kidney organogenesis by facilitating nephron progenitor cell proliferation.^67^ Furthermore, perivascular RMs closely interact with endothelial cells in developing renal vascularization.^68^ With such a strategic location, RMs monitor trans-endothelial transport of small protein/particles (ranging from 20 to 700 kDa or 10 to 200 nm) into the kidney interstitium.^69^ Small immune complexes (IC) that form by tagging microbial pathogens with immunoglobins fit within this size range and can be efficiently cleared by the kidneys (and not by the spleen and liver that would normally carry out such a function).^69^ In this way, RMs can promptly identify potential infectious particles, such as IC, and launch an immune response. Importantly, RMs act as sentinels in the kidney providing defense against bacterial infections from the urinary tract. RMs antimicrobial activity is performed cooperatively with neutrophils.^70,71^

#### 5. Liver macrophages

The liver acts as the central orchestrator of iron and lipid metabolism.^24,72,73^ Liver-residing macrophages are called Kupfer cells (KCs), which are embryonically derived and seeded along the sinusoidal endothelial cells^30^ that constitute sinusoids' walls. Hepatic sinusoids are formed by microvasculature arrangements to facilitate hepatic functions and maximize transport between the blood and the liver. At the sinusoids, oxygenated arterial blood mixes with portal vein blood carrying food nutrients/antigens from the intestine.^74–76^ The location of KCs enables them to scavenge and remove gut-derived pathogens from the blood stream. However, their clearance function goes beyond pathogens. KCs are also involved in removing damaged erythrocytes (red blood cells or RBC), and they tightly regulate iron metabolism.^24^ Furthermore, KCs gene expression profile shows an enriched lipid metabolism compared with other TRMs.^77^ Recently, KCs have been found to regulate cholesterol metabolism via their high fatty acid transporter CD36 expression.^78^ However, the precise roles of KCs in lipid metabolisms in the steady state still need to be elucidated.^24^

An additional liver-resident macrophage population residing in the hepatic capsule has recently been reported: the liver capsular macrophages (LCMs). They are phenotypically and ontologically different from KC. In the steady state, they are replenished from blood monocytes, and they are functionally responsible for sensing and responding to peritoneal bacteria.^79^ In such a manner, liver macrophages, positioned differently in the parenchyma, deal with blood–borne pathogens and intestinal microbes coming to the liver through the portal vein by KCs. LCMs sense peritoneal pathogens to prevent intrahepatic bacterial dissemination.

#### 6. Lung macrophages

The lung contains two main TRMs subsets based on their anatomical position and function: alveolar macrophages (AMs) and interstitial macrophages (IMs).^80^ AMs are seeded in the lungs during embryogenesis and self-sustain during adulthood in the steady state.^37,43^ AMs are the most abundant immune cells that monitor the lung's alveoli, the units in the lungs responsible for air exchange. Within the alveolar cavity, AMs play crucial roles in maintaining the balance of pulmonary surfactant (a phospholipid detergent that helps reduce alveolar surface tension, preventing their collapse and facilitating efficient gas exchange in the lungs)^81^ and eliminating dead cells (known as efferocytosis) fundamental for tissue homeostasis and organ function.^81^ Furthermore, AMs continuously patrol, phagocytose, and neutralize inhaled pathogens and particles without eliciting an excessive immune response through their tissue-specialized tolerogenic function (or immunoregulatory function).^82,83^ In such a manner, AMs modulate the immunological response that could cause tissue injury, thus preventing lung inflammation.^82^

IMs are located in the interalveolar septa and in proximity to the pulmonary vasculature acting as sentinels of the lung interstitium and the vasculature,^80^ promoting tissue repair.^11^ Also, they regulate the permeability of lung blood vessels.^11^ Such a population of PVMs in the lungs is monocyte-derived, long-lived, and slowly replaced by BM-derived monocytes in the steady state.^84^ Recently, a third class of IMs has been identified in close association with airway nerves.^11,85^ Their ontogeny remains controversial, with evidence pointing out a monocytic origin^11^ and contradictory studies suggesting an embryonic origin (YS-derived).^85^ Nerve-associated macrophages in the lungs show an immunoregulatory function^11,85^ and enhanced antigen-presentation capability compared to perivascular macrophages.^11^

#### 7. Skin macrophages

Macrophages are the largest population of resident immune cells in the skin with differently localized heterogeneous subsets.^86^ Human skin has two main layers: the epidermis, which is the outer stratus, and the dermis. Langerhans cells (LCs) are epidermal-resident macrophages and share many functional characteristics with dendritic cells.^87^ As a result of these similarities, LCs have only recently been recognized as TRMs since ontogenic studies revealed the embryonic origin of LCs from macrophage precursors.^88^ LCs positioned within keratinocytes (main cells forming the skin barrier) preserve barrier integrity by forming cell–cell tight junctions and constantly sampling for foreign antigens through membrane extensions called dendrites.^89^ Furthermore, LCs are potent antigen-presenting cells with good migratory capacity to lymph nodes and T-cell priming (antigen presentation to naïve T cells.^90^

Dermal macrophages consist of monocyte- and embryonic-derived macrophages residing in the dermis^41^ in different subcellular niches, located either adjacent to nerve bundles and fibers or blood vessels.^11^ They perform a scavenging role but show poor migration and T-cell activation capability.^41^ Nerve-associated macrophages actively patrol axons and promote nerve regeneration in both development and upon tissue damage.^86^ Instead, vessel-associated macrophages contribute to dermal blood vessel integrity at the steady state^11^ and coordinate neutrophil recruitment to inflamed skin during infection.^91^

#### 8. Spleen macrophages

The spleen is the largest secondary lymphatic organ that plays a crucial role in the immune system in addition to its functions in hematopoiesis (i.e., formation of specialized blood cells from progenitor cells)^92^ and clearance of exhausted red blood cells. Unlike other lymphatic organs, the spleen does not have afferent (inward) lymphatic vessels. Instead, it receives blood from the circulatory system, which brings in cells and antigens for immune cells to fight off foreign substances.^93^ Due to the anatomical differences between murine and human spleens, there are discrepancies in the classification of splenic macrophages according to their anatomical localization.^93^ In all three species, the spleen contains two regions: the white pulp and the red pulp. The latter comprises reticular connective tissue and contains all types of blood cells, including red pulp macrophages (RPMs). These macrophages phagocytose damaged and aged red blood cells and recycle iron.^75^ In the white pulp, tangible body macrophages also known as white pulps macrophages (WPMs) are localized within the germinal centers (GCs) alongside T and B lymphocytes.^94^ Within the GCs, B cells mature into antibody-producing B cells and memory B cells that mediate protection against future invading pathogens. During this process, WPMs are responsible for removing apoptotic B cell debris and modulating immune tolerance.^95^ RPMs derive from embryonic precursors while WPMs ontogeny has yet to be fully elucidated.^30,93^ However, a recent study has proposed the BM-origin of WPMs, stating that monocyte-derived precursors residing within the lymph nodes enter the GCs before the immune challenge resulting in resident WPMs.^96^

Given these insights into the functional specification of macrophages, it becomes clear that their purpose extends beyond their gatekeeping role. In addition to their immunological function, they exert key metabolic and physiological actions in their host tissues that are specifically adapted to keep organ operation and integrity [Fig. 1(b)]. It is important to further analyze macrophage biology, specifically how they acquire their core functions and whether their functional properties are determined by their ontogeny, due to the varying macrophage heterogeneity found in different tissues. It remains unclear how much ontogeny (embryonic and BM-derived TRMs) matters functionally in the steady state and how different developmental origins may affect macrophage response to disease signals. Indeed, macrophages sit at the interface of tissue homeostasis and pathogenesis. Any disruption in their core functions can lead to disease as well as contribute to it. Consequently, macrophages are potential targets for treating various inflammatory conditions. Targeting a specific macrophage phenotype could allow the selective manipulation of a given tissue without harming the host's immunity.

### B. Macrophages in disease

During steady-state conditions, the majority of tissue-resident macrophages (TRMs) arise from embryonic precursors that persist throughout an individual's lifespan. This occurs mainly through local proliferation and is not heavily dependent on the circulating hematopoietic system. However, in the case of tissue inflammation, a significant number of adult-derived monocytes enter the affected tissues and transform into macrophage-like cells. This process occurs simultaneously with the local expansion of TRMs, which is a slower and less energy-intensive mechanism.^97^ MDMs are a distinct population of macrophages that possess unique functional properties compared to other TRMs. TRMs are typically considered to have anti-inflammatory and reparative properties that contribute to tissue stability. In contrast, MDMs originate from blood monocytes and can transform into pro-inflammatory macrophages with microbicidal capabilities. However, these macrophages are also linked to an excessive inflammatory response that can result in harm to the injured or inflamed tissue. This belief is backed by solid evidence.^98–100^

When tissue homeostasis is disrupted by severe external damage, the resident macrophage population gradually decreases.^101,102^ As a result, monocytes from the BM are recruited to the inflamed tissue and differentiate into MDMs with a pro-inflammatory phenotype to handle the tissue injury or eliminate the pathogen. At the end of the inflammatory response, some MDMs disappear while others become residents.^22^ The steady tissue macrophage population is made up of TRMs present before the insult and newly recruited MDMs. The two populations are almost indistinguishable at the transcriptional level, with a correlation of over 98%.^103^ Notably, the gene Marco, which is exclusively expressed by embryonic-derived TRMs,^104^ remains unchanged by the environment.

**FIG. 2.**
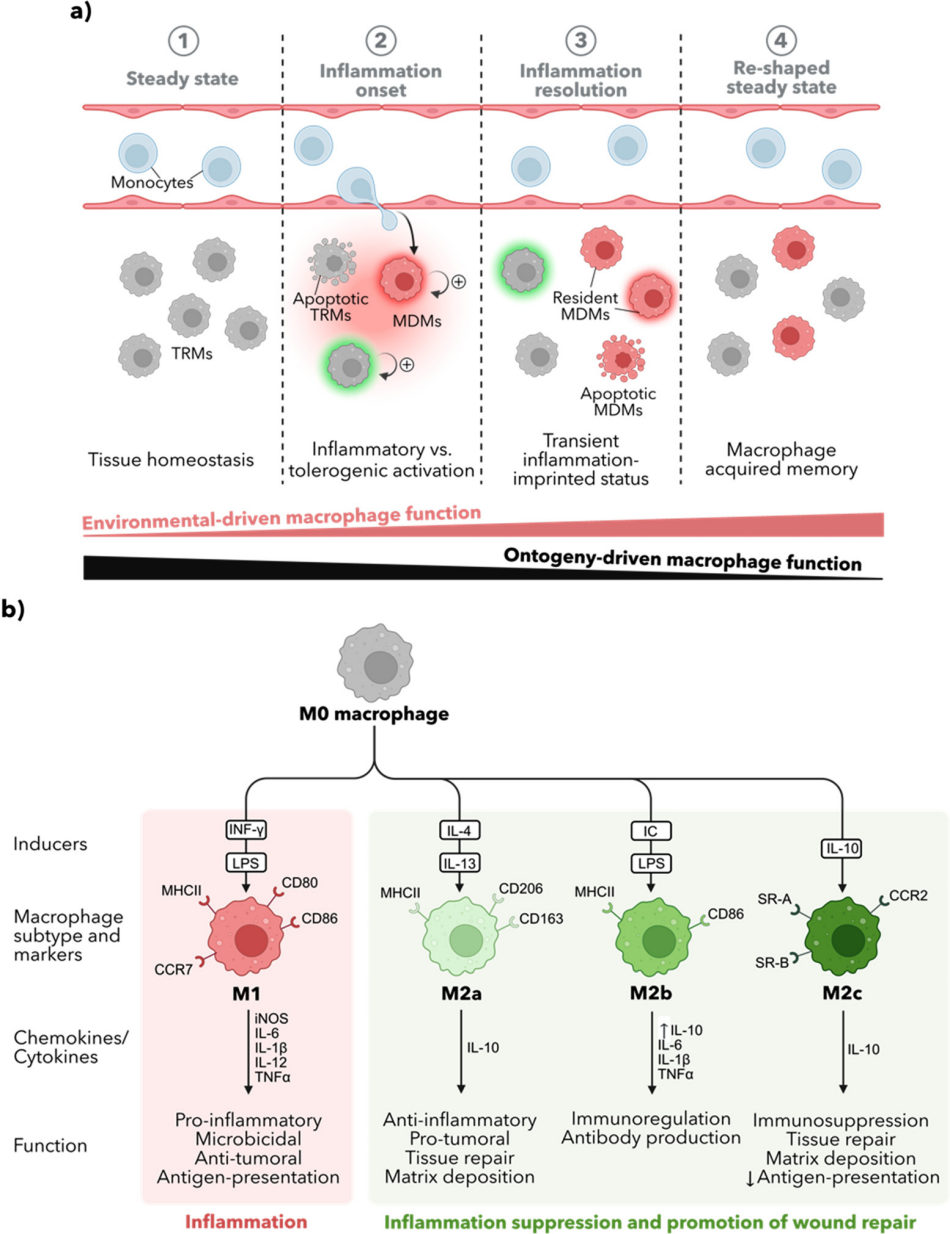
Macrophage activation in inflammation. (a) Inflammation reshapes the tissue macrophage pool. An inflammatory episode results in the recruitment of circulating monocytes from the blood vessels. They traverse the endothelium into the inflamed parenchyma and differentiate into monocyte-derived-macrophages (MDMs). This MDMs are distinct from the resident macrophages (regardless of their origin) as MDMs generally express a pro-inflammatory signature (red halo) resulting from the inflammatory environment in which they differentiated. Instead, tissue resident macrophages (TRMs) present before the insult possess a homeostatic imprinted identity that shapes their activation into a tolerogenic mode (green halo). The severity of the insult dictates the degree of macrophage depletion resulting in TRMs apoptosis. The macrophage population can be restored by local proliferation of both TRMs and MDMs. Upon inflammation resolution, the steady-state signals that maintain the TRMs profile gradually silence MDMs signature establishing a new population of resident MDMs. However, both TRMs and MDMs retain a transitory inflammatory signature, resulting in a stronger reaction to a second insult. Some monocytes differentiate into short-lived macrophages, which disappear as the inflammation in resolved. Once tissue steady state in reestablished, the tissue presents a re-shaped pool of resident macrophages with differently acquired immune memory. (b) Macrophage polarization. The functional classification of macrophages stablishes certain markers that characterize the acquired macrophage phenotype after stimulation of steady-state macrophages (M0) with different inducers. The M1/M2a classification or the so-called pro-inflammatory and anti-inflammatory polarization state of macrophages defines the functional role of M1 cells as capable of immuno-activation. Conversely, M2a cells promote immunosuppression for tissue regeneration. The M2 class of macrophages was later expanded to M2b and M2c cells.

Recent studies proposed memory genetic signature on MDMs with increased resistance to subsequent insults, whether of the same^105^ or different quality.^101^ For example, during acute influenza A virus infection (common respiratory pathogen), AMs were drastically depleted and became rapidly substituted by MDMs. After virus elimination and inflammation resolution, MDMs reside in the mice lung parenchyma as self-sustaining mo-ResAMs and monocyte-transcriptionally resembling. A month after the infection, post-influenza mice were significantly less susceptible to *Streptococcus pneumoniae* bacterial challenge than naïve mice. However, two months after influenza, the recruited MDMs became transcriptionally and functionally like resident AMs and no longer provided antibacterial protection.^101^ These results reflect the potential of macrophages to develop a “memory-like” protective immune response after infectious or noninfectious challenges, therefore determining a transient inflammatory-imprinted macrophage status [Fig. 2(a)].^106^

In line with this, induced respiratory infection with murid herpesvirus 4 (MuHV-4) in mice caused alveolar niche replacement of resident AMs by BM-derived AMs that conferred strong and lasting immune protection against experimentally induced asthma.^102^ Whether the conferred protection is related to AM origin (embryonic or BM-derived) or might be a consequence of the inflammatory environment caused by the infection remains unsolved. However, these results suggest that past inflammatory insults can remodel the lung immune system toward a trained protective state, although less specific than the one mediated by lymphocytes, capable of differentially modulating the outcome of future immune challenges. Complementary to these studies, the formation and maintenance of memory AMs have been demonstrated to be independent of BM-progenitors.^107^ Presumably, during a weak-intensity inflammation, the resident AMs depletion is scarce, and the remaining AMs can self-proliferate and repopulate the niche without contribution from circulating monocytes.^4^ When investigating *in vivo* the mechanism of trained innate immunity, researchers found that the priming (but not the maintenance) of memory AMs requires the help of effector T cells in an IFN-g- and contact-dependent manner.^107^ Interestingly, this observation establishes a new paradigm of immunological memory formation by linking adaptive immunity and innate immune memory in reversed order of classical adaptive memory formation by antigen-presenting cells.

Together, these observations highlight the importance of both tissue niche and cell origin in contributing to macrophage function upon disruption of homeostasis. However, the relative impact of cellular ontogeny vs tissue environment in establishing and maintaining tissue macrophage identity remains poorly understood. Evidence suggests the crucial role of the latest in determining macrophage responsiveness. An inflamed environment shapes macrophage-trained immunity, conferring increased responsiveness to secondary stimuli. Such response is pathogen unspecific, and it is mediated through cell epigenetic reprogramming leading to transient changes in cell physiology that persist for weeks to months after the elimination of the initial stimulus. Stroma-derived signals from the recovered microenvironment repress the pro-inflammatory phenotype and promote functional reprogramming of macrophages to re-establish tissue homeostasis [Fig. 2(a)]. In this way, multiple infections during life may, therefore, allow the acquisition of various macrophage populations with differently acquired memory.^4^

Furthermore, a highly plastic phenotype is frequently associated with MDMs. In other words, MDMs show higher susceptibility to microenvironmental stimuli responding with a marked change in their effector function. Conversely, a robust niche-imprinted tissue-specific phenotype is correlated with TRMs.^4^ Such an assumption requires further investigation as it is unclear how ontogeny shapes macrophage plasticity, and limited evidence sustaining such a statement is present so far. However, it is a fact that macrophage heterogenicity is augmented by the different activation states adopted in response to pathological stimulation, increasing the spectrum of possible macrophage phenotypes found *in vivo*. Researchers have provided themselves with an operational and simplified classification of the macrophage's activation state that defines two main polarization states. These are the so-called classical-activated or alternatively activated macrophages, also known as M1 and M2 phenotypes, respectively, emulating the T-cell nomenclature. Macrophage polarization refers to how macrophages have been activated in response to an environmental stimulus at a given point in space and time.^5^ Polarized macrophages differ regarding receptor expression, effector function, and cytokine and chemokine production.^6^

A pro-inflammatory signature characterizes M1 macrophages [Fig. 2(b)]. Its differentiation is initiated via interaction with bacterial components, such as lipopolysaccharide (LPS) and concomitant interferon-gamma (IFNγ) stimulation, inducing iNOS production, secretion of pro-inflammatory cytokines (IL-6, IL-1β, IL-12, and TNFα) to promote microbial killing, and CCR7 receptor expression involved in antigen presentation. In addition, M1 cells display high levels of major histocompatibility complex II (MHC-II) and co-stimulatory molecules CD80 and CD86^108^ for the activation of T cells important for the clearance of infected cells and the initiating an effective adaptive immune response. When a pathogen enters the body, macrophages phagocytose and degrade the pathogen, and then present fragments of the pathogen's proteins on their surface using major MHC molecules to T cells with concomitant CD80 and CD86 engagement, providing a second signal that is required for T cell activation.^109^ Functionally, M1 macrophages mediate the host defense eliciting a potent microbicidal role and anti-tumor immunity.^108^

M2 macrophages exhibit an anti-inflammatory phenotype. M2s are usually induced by stimulation with interleukine-4 (IL-4) and interleukine-13 (IL-13) and trigger the production of interleukine-10 (IL-10) with a critical role in limiting the duration and intensity of immune and inflammatory reactions.^108^ The representative function of M2 cells is inflammation suppression and promotion of wound repair.^3^ The bimodal classification was later considered extremes of a continuum of diverse functional states and further M2-like subtypes were introduced depending on the specific environmental stimuli: M2a (IL-4+IL-13), M2b (IC+LPS), and M2c (IL-10). While classic M2 macrophages are classified into M2a macrophages, M2b macrophages are induced by immune complexes (IC) with a concomitant low dose of LPS.^110^ M2b cells are not anti-inflammatory *per se* as they produce significant levels of TNFα, IL-1β, and IL-6 while a higher level of IL-10 production is observed compared to M2a cells.^108^ Functionally, M2b cells contribute to immunoregulation and promote antibody production.^111,112^ M2c cells are stimulated with IL-10 and glucocorticoids. They participate in immunosuppression, tissue repair, and matrix deposition and show limited antigen presentation capability.^108^

The categorizing of macrophages based on their polarization state is currently limited to *in vitro* models that rely on specific markers associated with known stimuli-induced polarization states. This approach is incomplete as it overlooks many other factors that contribute to macrophage behavior *in vivo*. A more comprehensive approach is needed to address this, one that considers the functional differences between homeostatic TRMs and MDMs and incorporates ontogeny. Such an approach would provide a more integrated conceptual framework and deepen our understanding of macrophage dynamics, which is crucial for better comprehending macrophage heterogeneity *in vivo.*

### C. The protective and the pathogenic role of different macrophage subsets in *M. Tuberculosis* infection

*Mycobacterium tuberculosis* (*Mtb*) is the etiological agent causing TB disease in humans and can establish a long-lasting, chronic infection by conquering and residing inside the very same cells that will typically eliminate it, a phenomenon known as the “macrophage paradox.”^14^ AMs are the most abundant host cells at the sites of infection^113^ and can recognize pathogens through an array of pattern recognition receptors (PRRs) and phagocytic receptors that are essential in the initiation of the innate immune response.^114^ Indeed, the early events of macrophage interaction with *Mtb* define subsequent infection progression and disease outcome.^115^ The study of macrophage heterogeneity is becoming fundamental for understanding their various roles during mycobacterial infection, either because of macrophages' specific activation phenotype or, more recently assessed, their developmental origin.

Throughout the airways, *Mtb* accesses the lungs and reaches the pulmonary alveoli. The existence of diverse macrophage subtypes in healthy lungs, both in terms of function and anatomical location, implies that these distinct phagocytes have varied contributions to immune surveillance. AMs are the primary resident defenders of the alveolus. While AMs can effectively eradicate routinely inhaled microbes, they fail to do so for host-adapted intracellular pathogens such as *Mtb*.^114^ As previously mentioned, AMs are FL-derived macrophages capable of self-renewal at a steady state^37^ with unique tissue-specific functions.^26^ AMs have an immuno-regulatory phenotype with a highly balanced pro- and anti-inflammatory response, fundamental for preserving the alveolar structure and its essential gas-exchange function.^83,116^ IMs are located in the bronchial interstitium and oppositely to AMs, most IMs arise from monocytes at a steady state.^84^ Macrophage heterogeneity found in the lungs before the introduction of *Mtb* infection suggests that the different phagocytes play different roles in promoting or controlling the infection. Indeed, AMs and IMs from uninfected mice revealed differential transcriptomic profiles under normal homeostasis and acquisition of distinct metabolic states. While IMs are highly glycolytically active, AMs are up-regulated for fatty acid uptake and β-oxidation^15^ suggesting that the divergence of response between AMs and IMs is epigenetically controlled and precedes mycobacterial insult. *Mtb* is well known to rely on host lipids metabolism for its intracellular survival^117^ suggesting a metabolic-permissive environment for *Mtb* within AMs present before infection. Such an increase in AMs susceptibility for bacterial growth has been experimentally validated, demonstrating that bacilli in AMs experience less stress and maintain higher replication activity than those present in IMs.^15^

Furthermore, the suboptimal response of AMs to *Mtb* is driven by the up-regulation of the transcription factor “Nuclear factor erythroid 2-Related factor 2 (NRF2).” NRF2 mediates a cell-protective antioxidant transcriptional program that explains the lack of response and delayed T-cell priming by infected-AMs in the early phase of infection.^118^ Interestingly, the up-regulated NRF2 expression is modulated by the lung microenvironment, suggesting a stroma-derived imprinting that shapes AMs function into a tolerogenic mode. This evidence supports the hypothesis that both cell-intrinsic and environmental factors imprint a tolerogenic mode in AMs, which impedes host control of bacterial growth at the early stage of infection. This, in turn, promotes *Mtb* infection onset to occur almost exclusively within resident permissive-AMs.^118^

Eventually, chronic infection is established in the lung interstitium driven by the selective migration of *Mtb*-infected AMs from the alveolar epithelium to the lung interstitium.^118^ *Mtb*-infected AMs relocation is dependent on host IL-1R signaling and the *Mtb* type VII secretion system, ESX-1 (also known as ESAT-6 secretion system 1), which is a major virulent factor of *Mtb*.^119^ At the lung interstitium, *Mtb* infects additional phagocytic cell types, including resident IMs and recruited MDMs. Indeed, IMs, MDMs, monocytes, and neutrophils are the most abundant myeloid cell types in the lungs following *Mtb* infection.^120^

According to a mice-infection model, it was observed that IMs are more efficient in controlling *Mtb* growth compared to AMs after 2 weeks post-infection (2 wpi). IMs displayed an M1 polarization state by actively producing pro-inflammatory cytokines IL-1β and TNFα and showed a lower bacteria replication rate. Furthermore, bacterial death was significantly higher within IMs in comparison to bacteria within neutrophils and AMs^15^ indicating a growing restrictive environment for *Mtb* in IMs. Transcriptomic analysis of human monocyte-derived macrophages further correlated a more robust inflammatory response to *Mtb* in IMs than AMs.^121^

A prevailing idea is that IMs are more restrictive than AMs. However, a recent study performed over longer stages of *Mtb* infection revealed that MDMs become the major intracellular reservoir for *Mtb* in the mouse lung (at 6 wpi) and have the highest rate of infection.^120^ However, the reason why *Mtb* preferentially adopts MDMs as a reservoir remains unsolved. A mechanism that involves the PGL-STING-mediated pathway of CCL2-mediated induction of monocyte recruitment by *Mtb*-infected AMs may explain how *Mtb* is capable of recruiting and establishing residence within grow-permissive monocytes. The mycobacterial membrane phenolic glycolipid (PGL) plays a critical role in activating the STING cytosolic sensing pathway in resident macrophages inducing the production of the monocyte chemoattract CCL2. Subsequent transient fusion of the infected macrophages and CCL2^+^ recruited monocytes enable bacterial transfer and dissemination of *Mtb* into a growth-permissive niche.^122^

With infection progression, *Mtb'*s ability to divert the host's innate immune response inhibits inflammation and induces an immunoregulatory phenotype in the host macrophages.^119^ In response to early *Mtb* infection, macrophages develop an increased glycolytic activity^123^ fundamental for the induction of an anti-microbial response.^15^ However, excessive glycolysis drives fatty acid synthesis and lipid accumulation in M1 macrophages, further differentiating toward the M2 immunomodulatory phenotype, which is closely linked to the virulent factor ESAT-6. The metabolic reprogramming of macrophages in response to *Mtb* infection, with a consequent dampening of macrophage pro-inflammatory and antimicrobial response, is hypothesized to precede foamy macrophage (FMs) differentiation.^119^ FMs are the product of an inflammatory response that drives macrophage conversion into foam cells through the dysregulation in the influx/efflux balance of low-density lipoprotein (LDL) from the serum.^124^ LDL particles contain cholesterol that is retained within lipid droplets in the cell cytoplasm.^125^ FMs differentiation happens at a later stage of infection and promotes a lipid-rich cell niche sustainable for *Mtb* growth.^119^

The M1–M2 repolarization,^126^ the FMs differentiation,^127^ together with monocyte influx^128^ drive granuloma formation. Granulomas are heterogeneous, dynamic, and spatially organized structures composed of macrophages, granulocytes, lymphocytes, epithelial cells, and fibroblasts.^129^ In response to persistent *Mtb* infection, granuloma formation is fundamental for bacilli containment within the myeloid core. This significantly limits the uncontrolled dissemination of infection to the uninvolved lung parenchyma. However, growing observations suggest that granulomas contribute to early bacterial growth, which is partially due to an upregulation of tolerogenic pathways within the region, thus resulting in a reduced bacterial clearance.^128,129^ Despite the diverse range of granuloma types, there appears to be a common spatial pattern in the organization of granulomas with different spatially encoded regulatory mechanisms.^130^ The central region of granulomas is macrophage-rich and possesses a pro-inflammatory environment with bactericidal activity. Conversely, the periphery possesses a comparatively anti-inflammatory signature with lymphocyte populations alongside immunoregulatory macrophages.^131,132^ Likely, such a spatial organization may limit the immunopathogenic antimicrobial activity toward those bacteria-enriched microenvironments keeping safely away the uninvolved tissue.

Russell and co-workers provided the first insight into macrophage ontogeny contribution to the interaction and outcome of initial *Mtb* infection.^15^ At the granuloma level, the lung local microenvironment together with cell–pathogen interactions driven by microbial virulence determinants, become the main modulators of macrophage function. Dynamisms of the macrophage activation state play a central role in determining granuloma fate. The local balance between anti- and pro-inflammatory cues affects the capacity of the host to resolve or isolate the pathogen during lung infection.^133^ However, many questions remain unanswered regarding the spatial organization of macrophage subsets in granulomas and whether microenvironment-specific inducers drive macrophage function into tolerogenic or bactericidal functions. This may be because the current research focus on granulomas is centered around investigating the environmental factors that influence macrophage behavior. As a result, the M1/M2 classification system is still used as a conceptual framework for understanding how immune signals affect macrophage activation. However, given the growing understanding of the role of myeloid cell development in determining macrophage function and its impact on TB pathology, it is necessary to complement current TB research with an assessment of the developmental origin of myeloid cells. The distinction between TRMs and disease-driven MDMs allows better characterization of the dynamics of MDMs recruitment and their functional contribution to the different stages of TB disease progression, as well as the functional relevance and degree of expansion and replacement of TRMs. Enhanced comprehension of this dynamic would complement the characterization of macrophage heterogeneity in the context of *Mtb* infection, as macrophages remain a crucial therapeutic target for microbicidal strategies. This would aid in guiding therapeutic intervention at various stages of diseases, offering a chance to target pathogenic macrophage programs in a tissue-specific and disease-specific manner with greater precision.

Creating effective targeting systems relies on multivalent L–R interactions exploiting differences in receptor density. By engaging various weak interactions, they achieve a selective binding onset.^134^ This approach draws inspiration from common multivalent interactions in biological systems and has found extensive use in nanomedicine for targeting purpose, as we delve into later. A notable aspect of multivalent binding is “superselectivity,” allowing the differentiation of surfaces based on their relative receptor densities.^135^ Consequently, properly designed multivalent systems targeting different receptor types can specifically bind a chosen receptor density profile, providing a mean to target cells without relying on a single dominant marker, which is generally challenging to identify.

## III. MULTIVALENT INTERACTIONS

The immune system is equipped with different mechanisms to recognize and destroy pathogens. The success of the recognition is dependent on the multivalent association of ligand–receptor (L–R) pairs between the invading organism and specific receptors displayed on sentinels cells, mainly macrophages. Such an immediate processes is known as the innate immune system.^136^ Macrophages represent the main effector cells in the innate immune system, bridging the innate and the adaptive immune systems with their antigen presentation role.^137^ The adaptive immune system—led by lymphocytes, B cells, and T cells—performs an antigen-specific recognition and ensures long-lived immunological memory against reinfection.^136^ After macrophage pathogen recognition, multi-meric L–R associations lead to pathogen internalization, which is further processed into smaller fragments called antigens.^138^ The antigens are then displayed on the surface of the macrophage bound to specialized molecules (MHC molecules), a process known as antigen presentation. The presented antigens on macrophages serve as signals to stimulate B and T cells. This process involves the interaction of APCs' MHC molecules with loaded antigens and the corresponding T cell receptor (TCR) recognizing the presented antigen. To initiate the signal transduction through the TCR, the antigen-MHC ligands form multivalent complexes that adopt dimeric or trimeric conformations, thereby increasing binding stability.^139^ Antigen-MHC complexes may be present in low numbers. Because of the relatively low affinity of the TCR–ligand interaction, sustained conjugate formation between T cells and APCs is necessary for T cell activation.^140^ Thereby, adhesion molecules interact with their ligands on the opposing cell surface, like integrin (LFA-1)-intercellular adhesion molecule–1 (ICAM-1) binding, stabilizing the interaction.^141^ In such a manner, multiple ligands-receptor pairs bind simultaneously to enhance binding selectivity and avidity while prolonging the duration of the interaction. Stable binding is essential for signal transduction, inducing immunological function and regulation.^142^ As a result, B cells produce antibodies (specific antigen-binding proteins that neutralize the antigens or mark them for destruction by other immune cells). In contrast, T cells help regulate the immune response and eliminate the foreign antigen.^136^

Similarly, initial pathogen recognition by macrophages requires forming a stable adhesion to finally trigger their activation. The immune system possesses various mechanisms that enable it to detect and selectively recognize foreign microorganisms. The complement system (CS) is one of the key components of innate immunity mediating pathogen recognition. The CS is made up of many distinct soluble plasma proteins called opsonins. They recognize well-conserved antigens, i.e., commonly bearing molecules like bacterial membrane components, and bind to them to tag pathogens for destruction, process known as opsonization. Phagocytic cells recognize opsonized pathogens by expressing membrane complement receptors (CRs).^143^ Similarly, opsonizing immune antibodies (Abs) recognize pathogens via their specific epitope and use their Fc domain to interact with receptors (FcRs) highly expressed in macrophages.^144^ While the CS provides immediate-nonspecific recognition, Abs offer specific and targeted recognition of particular antigens. However, both mechanisms utilize multivalency due to their molecular structure and mechanism of engagement and can act concomitantly to recognize pathogens through opsonization.

The overall topology of Abs is highly conserved and they are the quintessential example of multivalency. The multivalent property of Abs is augmented by the conformation that Abs adopt, which is related to their valency and affects how a single Ab molecule interacts with the antigen-bearing surface. Ab valency, i.e., the number of individual connections of the same kind that can be formed through L–R interactions,^145^ varies across different Abs classes. Five mammalian immunoglobins classes arise from the combination of a Y-shaped monomer comprising two antigen binding sites and one Fc domain.^146^ IgG, IgD, and IgE are monomeric, thus bivalent toward the antigen and monovalent toward the FcRs. IgA is typically a dimer, and IgM is typically a pentamer, with both also reported to form other association states including tetrameter and trimer.^146,147^ IgM is also found in their monomeric state on the surface of B lymphocytes as membrane-associated antigen-recognition receptors.^148^ When oligomerized, stellate-like IgM pentamers acquire distinct functionalities compared to their monovalent counterparts. Soluble pentameric IgM shows a remarkable ability of pathogen neutralization^149^ potentially through their flexible antigen binding domains adopting a dome-shaped arrangement on the antigen-bearing surface.^150,151^ Furthermore, the stellate to dome-shaped transition exposes complement-opsonins regions allowing C1q binding and resulting in an IgM-C1q complex that triggers CS activation.^151^ Therefore, higher-order oligomers provide an avidity advantage through their multivalent binding and engagement of the CS, converting relatively low-affinity interactions into combinatorial high-avidity associations that enable selective pathogen recognition and neutralization.

Abs exert an immune antimicrobial activity via Fc domain engagement with FcRs found on all innate immune cells.^142,152^ The interaction of low numbers of Fc domains with FcRs is too weak to induce macrophage phagocytosis. However, when multiple antibodies are bound to a pathogen, the host cell membrane advances over the Abs-opsonized bacteria, sequentially engaging multiple FcRs on the surface of the macrophage. After exceeding a density threshold of receptor engagement, the interaction is strong enough to stabilize the cell–pathogen contact through multivalent interactions enabling successful internalization.^142^

Opsonin receptors (CRs and FcRs) recognize host-derived opsonins bound on the target. Additionally, macrophages are equipped with non-opsonin receptors that bind to specific molecular motifs present on the surface of bacteria. Different types of receptors are engaged depending on the pathogen, and several examples of bacterial infection have been nicely reviewed and listed in the past.^145^ In TB infection, the *Mtb* bacilli are inhaled and then engulfed by AMs through a process called phagocytosis. The recognition of the pathogen by AMs involves the activation of pathogen recognition receptors (PRRs), which can identify highly conserved molecular structures present on the surface of pathogens.^153^ These structures are called pathogen-associated molecular patterns (PAMPs) such as glycolipids, phospholipids, and lipoproteins distributed within the different layers forming the bacteria cell wall. Several families of PRRs exist, including membrane-bound and intracellular Toll-like receptors (TLRs) and cytosolic NOD-like receptors (NLRs), RIG-I-like receptors (RLRs), C-type lectin receptors, and scavenger receptors. Each PRRs family binds to a different repertoire of PAMPs, generating combinatorial signals resulting in macrophage activation.^114,154^ For example, the mannose receptor CD206 (or MRC1) is a C-type lectin receptor expressed on human AMs that binds to glycolipids lipoarabinomannan (LAM) and mannosylated LAM (ManLAM), present in the outer layer of the mycobacteria cell wall.^155^ Thereupon, *Mtb* engagement with CD206 induces an anti-inflammatory response.^156^ Conversely, TLR-mediated recognition of *Mtb* generally initiates an intracellular pro-inflammatory signaling cascade via TLR2 in association with TLR1/TLR6, by TLR4, or by TLR9.^157^ The type of macrophage activation (M1 or M2) that different engaged receptors induce is generally inherent to the receptor structure, bearing conserved cytosolic domains that trigger inflammatory intracellular cascades that lead to pro- or anti-inflammatory responses. For example, TLRs bear the Toll–IL-1 receptor (TIR) cytosolic domain, which activates common pro-inflammatory signaling pathways, most notably leading to the activation of the transcription factor NF-κB.^158^ In the case of the CD206 receptor, an intracellular signaling domain has not been identified.^158^ However, *Mtb* engagement with the CD206 receptor induces PPAR-γ nuclear expression that negatively regulates macrophage activation.^156^

Both mechanisms, opsonic and non-opsonic receptors, engage cooperatively for the recognition of pathogens.^159^ Collective effects strengthen the overall cell–bacteria interaction by enhancing the association avidity, i.e., the accumulated strength of multiple affinities.^160^ Furthermore, combinatorial low-affinity interactions and varied receptor engagement allow for macrophage superselective recognition of the pathogen molecular pattern, critical for initiating an immune response. The effect of multivalent association extends beyond the stability and selectivity in the recognition, allowing the initiation of specific intracellular signaling cascades that drive macrophage activation.^18,145^ Numerous pieces of evidence underscore the significant impact of spatiotemporal patterns on the extent of immune activation.^161^ Immune signaling is strongly influenced by crystalline properties such as the inter-ligand spacing and the domain size of the ordering as demonstrated in the work of Wong *et al.* employing DNA nanotechnology.^162,163^ For example, when organized crystalline arrays of double-stranded DNA (dsDNA) are presented, it triggers a notable increase in the secretion of IFN-α through TLR9 signaling in plasmacytoid dendritic cells. This enhanced signaling pathway is only observed when the spacing between DNA molecules aligns with the size of TLR9 receptors, allowing for the effective interlocking of multiple receptors and ligands.^162^ Similarly, the presentation of ordered lattices of dsRNA leads to amplification of TLR3-mediated IL-6 cytokine production in primary neonatal human epidermal keratinocytes (NHEKs),^163^ suggesting that both valency and induced organization of immune ligands into optimal periodic structures can result in massive binding enhancement, and thereby strongly amplify immune response.^161^ It is evident that immune modulation is a multifactorial process where physical mechanisms (multivalent interactions) play a crucial role in biology of the immune response. Starting from the initial recognition of pathogens, multiple receptor engagement allows their precise recognition as exogenous organisms.^159^ Subsequently, the consequences of these multivalent interactions manifest as heightened binding specificity, enhanced receptor recruitment, and the subsequent amplification of intracellular signaling cascades downstream. It can be hypothesized that like PRRs transmembrane receptors that induce specific signal transduction, multimeric associations may induce different signaling pathways able to govern cellular responses depending on several factors, such as the receptors involved, the arrangement of those receptors on the cell membrane, and the strength of the interaction. Hence, if a multivalent ligand induces the clustering of certain receptors on the host cell membrane, it may activate a particular signaling pathway that leads to a specific cellular response. Alternatively, if such interaction is weaker, it may induce a different set of signaling pathways, resulting in different signal transduction.

Shifting our perspective to the infective organism seeking a host, viral infection of a host cell represents another notable example of multivalent interactions within biological systems. In this scenario, the viral particle serves as a discerning system with the capability to infect a particular (or not) type of targeted cell. Viral particle attachment to the host cells starts with virus recognition and binding to multiple cell surface receptors via protein/protein interactions. The virus expressing multiple surface viral proteins forms a multivalent scaffold for selective binding of a target cell bearing a specific repertoire of receptors. The affinity between a single viral protein and receptor is dictated by nature. When combined into multivalent and multiplexed (i.e., the interaction that involves a mixture of L–R pairs) association profiles, these interactions create a sharp response to gradients of receptor density, resulting in superselective binding to a targeted cell phenotype.^19,134^

One aspect of the interaction between cells and viruses that is often overlooked is the initial interaction mediated by glycans. Glycosylation is a biochemical process that involves attaching a chain of sugar molecules (glycan) to a protein or lipid, leading to the formation of glycoproteins, proteoglycans, or glycolipids.^164^ When dendritic chains form on specific sites on protein surfaces, glycoproteins are formed, while proteoglycans have linear and long chains that form a cushion of carbohydrate chains that cover the cell membrane, known as the cell glycocalyx. Proteoglycans comprise a core protein with one or more covalently attached glycosaminoglycan (GAG) chains. Many viruses utilize GAG as attachment factors for host cell entry.^165^ The emergence of the coronavirus disease (COVID-19) has generated a great deal of interest in the mechanisms of viral infection, particularly in how the virus enters human cells. COVID-19 is caused by the severe acute respiratory syndrome coronavirus 2 (SARS-CoV-2). The spike glycoprotein (SPG) binding to cell membrane GAGs is the first step in successful host cell invasion of SARS-CoV-2 followed by engagement with several cellular-entry receptors (e.g., neuropilin,^166^ SR-B1,^167^ and ACE2^19^). Recently, a model that predicts the viral tropism of coronaviruses infecting ACE2-expressing cells has been proposed by our group. A key component of the model is considering both the entry receptor glycosylation and the cell proteoglycans in describing the cell–virus interaction. One of the most interesting outputs of the model is the role of the binding affinity of the envelope protein SPG toward heparan sulfate (HS, the predominant GAG in healthy lungs) and the ability of SARS-CoV-2, SARS-CoV, and MERS-CoV to bind to cells with different HS density. The model explains the clinically observed preference of SARS-CoV-2 to bind to the upper airways (high HS density) instead of other coronaviruses binding to lower airways (low HS density regions). In other words, the model describes how viral infection selectively targets a cell population with a specific receptor and proteoglycan composition.^19^ Nature tightly regulates the affinity of a single viral protein to key components of the cell membrane and the model explains how viral selectivity is based on the SPG's avidity toward. Additionally, viral particle size is finely tuned by natural selection to ensure host cell entry.^19^ Therefore, viral particles can be seen as highly efficient and sophisticated multivalent machinery that has evolved to facilitate the entry of their genetic material into host cells. The surface of a virus particle, covered with numerous copies of viral proteins, binds selectively through finely tuned avidity to a targeted cell population, whose phenotype is dictated by receptors and glycans expression involved in the association.

Is it possible to draw insights from the sophisticated mechanisms used by viruses for cell entry and the selective pathogen recognition exhibited by macrophages when designing systems that can specifically bind to particular cell populations? It is evident that certain biophysical factors must be considered when designing multivalent systems, including factors such as valency, topology, and combination of multiple L–R pairs. In the following section, we will explore the principles of statistical mechanics governing multivalent L–R interactions. We will also review recently proposed theoretical model systems that can help decouple the relevant parameters governing selective interactions. These models can offer valuable insights into predicting the optimal level of functionalization required for targeting specific cell phenotypes.

### A. Engineered multivalency

**FIG. 3.**
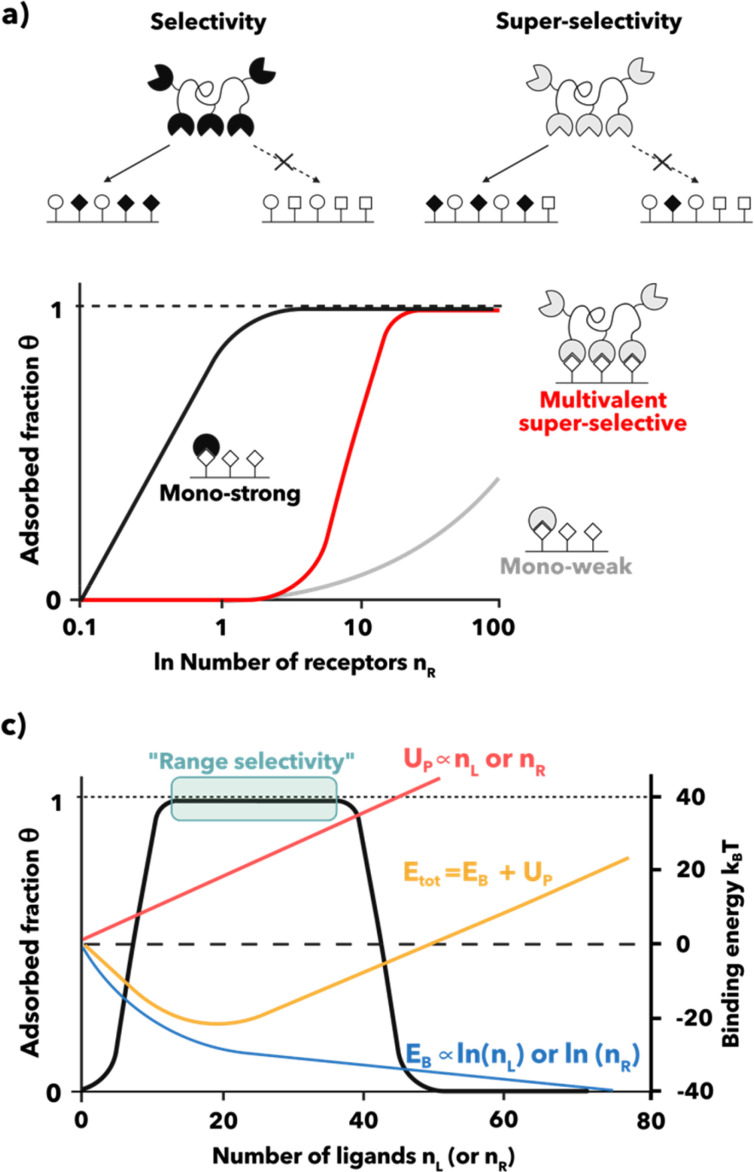
Selective systems. (a) Selectivity vs superselectivity. Conventional selectivity relies on high-affinity binding to specific receptors found only on diseased cells. However, strong binding affinity leads to a reduced concentration of the receptor needed to saturate the receptor bearing surface ( θ=1) that leads to nonspecific adhesion to both healthy and diseased cells, reducing the selectivity. Superselectivity, on the other hand, requires nanocarriers with low-affinity ligands that interact weakly with receptors. These nanocarriers rely on multivalent interactions and only bind strongly under conditions of high avidity. As a result, there is a sharp, sigmoidal change in receptor as the number of receptors increases. (b) Range selectivity. Ideally, the binding free energy (E_B_) of a multivalent system grows logarithmically with the number of ligands (n_L_) or receptors (n_R_). However, the topology of the system imposes a source of repulsion that has a linear dependence with the system valency (n_L_) or n_R_. Both contributors, the association force and the steric, result in a regime of maximal total binding energy (E_tot_) where association is enabled. Such binding regime is delimited by a lower and an upper threshold of n_L_ (or n_R_) that we dub range selectivity.

In nanomedicine design, multivalency plays a crucial role. Targeting systems are usually functionalized with multiple copies of ligands, creating an ideal model that mimics natural systems. Multivalent systems can form a strong interaction with targeted surfaces, but more interestingly, this interaction can be superselective, which sets itself apart from conventional type selectivity. Conventional selectivity distinguishes surfaces based on their unique receptor types. Such systems typically utilize high-affinity binding domains to a targeted protein that is solely expressed on diseased cells, which is not always readily available.^168^ While specific proteins may be up-regulated or uniquely expressed in certain diseases, it is only sometimes the case making it challenging to find a universally applicable target. When targeting systems are functionalized with high-affinity binders, a strong binding affinity leads to a reduced concentration of the receptor needed to saturate the guest [Fig. 3(a)]. Hence, if the target protein is moderately present in healthy cells, high affinity targeting agents will adhere to both diseased and healthy cells, causing a loss of selectivity.^134^ Such promiscuity is the major reason for drug off-target causing adverse side effects. Instead, superselectivity requires multivalent weak supramolecular interactions, such as weak receptor-ligand affinities. Targeting systems designed with low-affinity ligands require multivalent interactions that enable interaction exclusively under high avidity.^169,170^ This results in a typical sharp, sigmoidal change in the receptor saturation (which represents the quantitative expression for absorption) as a function of the number of receptors, a phenomenon known as superselectivity.^135,171,172^ In other words, binding occurs only over a certain threshold of receptor density, at which binding increases monotonically until receptor saturation. Since multivalency relies on the principle of “strength in numbers,” it might be tempting to adopt a “more is better” strategy when it comes to functionalizing nanocarriers with ligands. However, at higher ligand (or receptor) densities, the interaction becomes less favorable, which hinders the adsorption. The reason behind this can result counterintuitive. Briefly, the decline in interaction beyond a specific ligand (or receptor) density threshold is a consequence of the scaling of repulsive contributions due to excluded volume interactions. This results in a non-monotonic behavior termed as range selectivity.^172^ Range selectivity defines both a minimum and maximum average receptor density value within which binding is enabled. More insights into this concept will be provided hereinafter.

In sum, multivalency based on low-affinity ligands greatly increases the sensitivity of the targeting system to the surface receptor concentration within a specific range of receptor density. This represents an appealing strategy for biomedicine for discriminating different cell types.^135,160,172,173^ We present a theoretical analysis of the multivalency effects of polymer-based nanoparticles (NPs) functionalized with many ligands interacting with cells. The polymeric nature of the synthetic system allows the manipulation of several critical aspects of the interaction, such as NP size, ligand number, and accessibility, which collectively impact the multivalent binding effect.^174^

#### 1. Multivalent adsorption

**FIG. 4.**
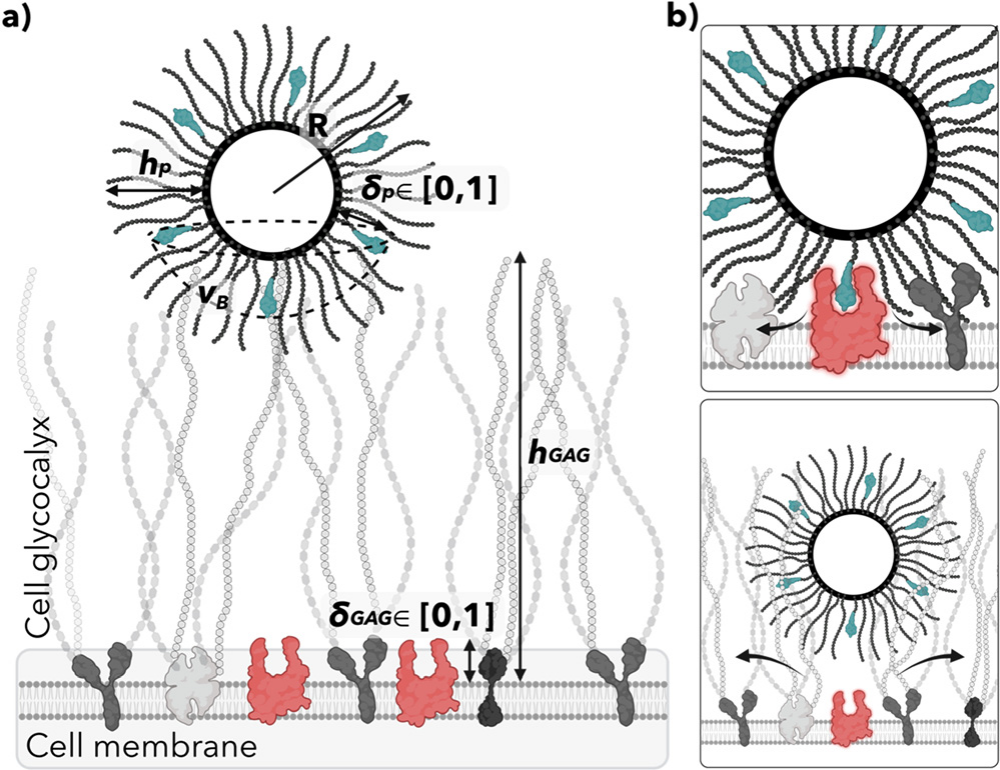
Nanoparticle (NP)–cell interaction. (a) The multicomponent association. The multivalent system is described by the NP topology, size, number of ligands, and length of the ligand tether with respect to the polymer brush length. The target, the cell, is relatively larger that the NP, hence considered as a flat surface with a density of receptors that characterizes the cell phenotype. (b) Sources of steric. Two sources of repulsion are opposing the attractive energy given by the ligand–receptor affinity. At the single-molecule level, excluded volumes interactions emerge from the polymer brush when the ligand approaches the receptor tip giving rise to a polymer steric. Preceding the polymer steric, the bulk NP has to overcome the repulsion from the cell glycocalyx. Formed by long sugar chains coating the cell that the NP displaces as it approaches the receptors. NP effective binding to the cell requires high avidity associations that can overcome the repulsive forces.

First, let us delve into the physics of multiple interactions and how combinatorial bonds can lead to associations driven purely by entropy. We define NPs by size, the number of functionalized ligands, and bulk concentration in solution. Conversely, the targeted surface (the cell) comprises several copies of the relative receptors on a considerably large surface, effectively flat.^19,173^ In such a scenario, the L–R interaction is the driving force for the association [Fig. 4(a)].

The Langmuir adsorption model provides a simple theory for molecular interactions at interfaces. Such a model is now one century old and has widespread applicability, including protein binding, further developed by Hill (Box 1).^175^ The fraction of the occupied surface by particles is described by Eq. (1) which depends on two variables, the nanoparticle activity and the grand canonical partition function. The former describes the binding area that the NP can explore relative to the concentration. The partition function describes the energy distribution among the different possible states where the multivalent unit is bound to the surface.

Box 1. Langmuir–Hill theory of absorption

Assuming independent adsorption sites and at most a single particle per site, we can use the well-known Langmuir–Hill absorption model^175^ to describe the experimentally observable cell surface coverage by the NPs, θ, as

$$\theta =\left\langle \frac{aQ_{NP}}{1+aQ_{NP}}\right\rangle ,$$ (1)

where a is the NP activity within the binding volume, $v_{B}$, i.e., the volume a particle can explore while bound [Fig. 4(a)]. NP activity can be approximated as $a\approx [NP]N_{A}v_{B}$, where [NP] is the molar concentration of the NP in the bulk solution and $N_{A}$ is the Avogadro number. The binding volume can be derived for a sphere with radius R. The angle brackets ⟨⟩ represent an average over all possible receptor numbers distribution weighted by their Poisson distribution.

Q is the partition function of the system and is the central quantity describing the strength of binding between a single NP and the cell surface. $Q_{NP}\left(N_{L,\;}N_{R},\beta {\Delta G}_{i}^{0}\right)$ is a function of the number of ligands $N_{L}$ on the nanoparticle, the number of receptors $N_{R}$ on the targeted surface (i.e., the cell), and the strength of the ligand–receptor bond, ${\Delta G}_{i}^{0}$, where the β=1/kT with *k* representing the Boltzmann's constant and *T* is the absolute temperature. *kT* is a widely use reference unit of energy convenient for describing molecular-scale systems.

In a multivalent system, two main factors contribute to the free energy of the complex. The first factor is the binding free energy of the initial interaction between the ligand and receptor. This binding free energy can be further broken down into two components [more in depth in Box 2, Eq. (2)]. The second factor is the combinatorial effect of multiple L–R complexes that occur subsequently. This effect, known as degeneracy, is unique to multivalent systems and refers to the number of possible binding states that the available ligands and receptors can adopt.^176^ The degeneracy factor gives rise to combinatorial entropy that favors the multivalent association and represents the major determinant of the free energy increase in multivalent associations in respect to the monovalent counterpart.^168,176^ Its magnitude becomes substantial as the receptor surface density (or ligand density on the NP) increases. The association of multivalent superselective systems sharply and non-linearly increases depending on the number of ligands present (Fig. 3). Multivalent interactions are characterized by the number of potential binding states, which is highly dependent on the abundance of ligands and receptors. The valency and the number of ligands while the receptor composition is given by the cell phenotype we want to target. We can, thus, adjust the multivalent associations by weakening the individual L–R bonds. This allows for selective targeting, where binding occurs through the combination of enhanced complex binding strength (avidity). By doing so, we can achieve superselective associations to unique receptor densities.^134^

Box 2. Free energy of bond formation

At the level of single ligand–receptor (L–R) interactions, the binding free energy of *i*th bond, can be written as

$${\Delta g}_{i}^{0}={\Delta G}_{\text{mono}}^{0}(i)+{\Delta G}_{\text{interaction}}^{0}.$$ (2)

It is convenient to separate the initial binding event ${\Delta G}_{\text{inter}}^{0}$, equivalent to the intrinsic monovalent interaction ${\Delta G}_{\text{mono}}^{0}$, from subsequent binding events that can occur on the remaining binding sites on the receptor surface, giving rise to differences in the successive intramolecular binding events, denoted as ${\Delta G}_{\text{intra}}^{0}$. Then, the collective binding energy of the complex (multivalent scaffold) RL(i) is found as

$${\Delta G}_{i}^{0}={\Delta G}_{\text{inter}}^{0}+\left(i-1\right){\Delta G}_{\text{intra}}^{0}-RT\;ln{\;\Omega }_{i}.$$ (3)

The latest equation denotes the contribution of the single ligand–receptor event defined by the ${\Delta G}_{\text{inter}}^{0}$ and ${\Delta G}_{\text{intra}}^{0}$ free energies, and the combinatorial effect of an ensemble of multiple bonds. Kitov and Bundle^176^ introduced the degeneracy coefficient $\Omega_{i}$ to describe the numerous ways equivalent bound forms may be achieved. This purely entropic term is unique to multivalent interactions and measures the degree of disorder in the distribution of L–R complexes. Its magnitude depends on the topology of the multivalent scaffold, in other words, how the ligands are presented to the receptor-bearing surface, as follows:

$$\Omega_{\left\{\text{indifferent}\right\}}(i)=N_{L}N_{R},$$ (4)

$$\Omega_{\left\{\text{linear}\right\}}(i)=(N_{L}-i+1)(N_{R}-i+1),$$ (5)

$$\Omega_{\left\{\text{radial}\right\}}(i)=\frac{N_{L}!N_{R}!}{\left(N_{L}-i\right)!\left(N_{R}-i\right)!i!}.$$ (6)

In Eq. (6), the degeneracy is maximized as it comprises multiple long and flexible tethered ligands symmetrically distributed on a core that render them equally accessible to each receptor subunit. Assuming that each ligand can only bind to one receptor, i.e., each ligand–receptor pair is independent of the rest. The partition function of a multivalent system can be expressed as

$$Q=\sum_{i=1}^{\text{min}\left(N_{L},N_{R}\right)}\;\Omega \left(i\right)e^{-i\beta {\Delta G}_{i}^{0}},$$ (7)

which can be easily transformed to get the full expression of the NP partition function by combining Eqs. (6) and (7) as

$$Q=\sum_{i=1}^{\text{min}(N_{L},N_{R})}\;\frac{N_{L}!N_{R}!}{\left(N_{L}-i\right)!\left(N_{R}-i\right)!i!}e^{-i\beta {\Delta G}_{i}^{0}}.$$ (8)

Equation (8) represents the partition function of the multivalent system considering all possible binding states denoted as *i*.

We proposed using polymer-based nanoparticles,^19,171,172^ known as polymersomes, as a strategic approach to maximize the degeneracy term in multivalent systems. Polymersomes have the advantage of adopting a radial topology, which enhances the potential for multiple L–R binding interactions. Moreover, the polymeric nature of these synthetic nanoparticles provides us with control over critical parameters such as nanoparticle size, composition, ligand number, and ligand accessibility (Fig. 4). These factors collectively contribute to the overall multivalent binding effect.^174^ Nanoparticles are constructed using a polymeric brush as a protective barrier against nonspecific interactions. Embedded within this brush are tethered ligands, which consist of flexible polymeric chains shorter in length than the brush itself. This design allows us to manipulate the accessibility of the ligand precisely, directly influencing its binding affinity. This tunability of ligand affinity is crucial for achieving superselective associations, addressing a significant limitation in applying the superselectivity theory.^171^ By implementing this engineering strategy, we can simultaneously address two challenges. First, the prevention of the formation of a protein corona. Second, the modulation of bond-mediated-specific interactions enables us to fine-tune and optimize the binding properties of the NPs.

#### 2. Sources of steric repulsion

In the context we are discussing, which involves polymer-coated nanoparticles housing embedded ligands, an important aspect to consider is the emergence of a steric repulsion force due to the presence of the polymeric brush. This force is applied by the polymeric chains to the receptor's tip as the ligand binds to its corresponding receptor, a situation visually represented in Fig. 4(b). This steric repulsion acts as a counterforce opposing the energy of association driven by the affinity between the ligand and receptor, effectively diminishing its strength. Such a setup offers a means to fine-tune the affinity between the ligand and receptor by controlling ligand accessibility, which is crucial for achieving a superselective behavior. However, such a configuration comes at a cost. As successive L–R associations occur, the steric repulsion exerted by the polymer increases linearly with the number of ligands, as shown in Fig. 3(b), eventually surpassing the attractive forces, and turning off the association. This gives rise to a range of binding free energy that determines the ideal NP valency needed for NP association, known as the “range selectivity regime.” Within this regime, there exist both lower and upper limits. The lower limit represents the minimum number of L–R associations necessary to initiate NP binding. If the number of associations falls below this threshold, binding cannot occur. On the other hand, the upper limit is determined by the maximum number of L–R associations, beyond which the repulsive forces outweigh the attractive ones, leading to the inhibition of association. The non-monotonic behavior of the range selectivity explains the redundancy in the design of high avidity systems, and, even more intriguingly, their ultimate inhibition of association.^172,177^

The confluence of multivalent attractive interactions coupled with steric effects, each exhibiting distinct dependencies on the receptor/ligand quantity, yields a non-monotonic trend. This trend's breadth and magnitude are modifiable through the manipulation of affinity, steric contributions, and binding volume. In effect, the selectivity can be engineered *a priori* by employing appropriate molecular design strategies.^172^

Until now, we have solely focused on the situation where binding is driven by the formation of L–R bonds and where steric hindrance occurs at the level of L–R interactions. This holds for numerous biological systems, particularly those involving specific binding.^172^ However, we must also consider the repulsive force experienced by the bulk NP as it approaches a cell membrane. This repulsion arises from excluded volume interactions caused by the cell glycocalyx, a polymer layer that covers the cell surface. For similarity, it can be derived as the polymeric steric, in depth as in Box 3. The final expression of the partition function describing the multivalent system can be expressed as in Eq. (12), making evident the way steric sources act differently on the system. The polymeric repulsive steric, *U_P_*, acts at the level of each L–R pair configuration. Conversely, the glycocalyx repulsive steric, *U_GAG_*, influences the bulk NP as the steric penalty affects the partition function of the entire system, Q (i.e., the partition function of a NP), making its binding less energetically favorable (Box 4).

Box 3. Steric potentials

When a NP comprising embedded ligands within a polymer brush coating approaches a targeted cell, the L–R interaction is the driving force for the association. The latter is countered by two sources of steric potentials: one originating from the cell glycocalyx and the other from the polymer brush coating the NP [Fig. 4(b)]. Steric potential refers to the repulsive forces between molecules that arise from their spatial arrangements and excluded volumes between molecules that are approaching.^172^ The steric potential signifies the minimum energy threshold that multivalent NPs must surpass to overcome repulsive forces, thus facilitating their association.

As the nanoparticle (NP) approaches the binding site, the receptor experiences repulsion from the NP's polymer brush chains, resulting in what we refer to as polymeric steric energy, $U_{P}$. This repulsive free energy affects each single ligand–receptor bond formation. Hence, Eq. (8) can be re-written as

$$Q=\sum_{i=1}^{\text{min}\left(N_{L},N_{R}\right)}\;\Omega \left(i\right)e^{-i\beta {(\Delta G}_{i}^{0}+U_{P})},$$ (9)

where $U_{P}$ is the steric potential arising from the receptor inserting in the NP brush and displacing the polymeric chains, giving rise to osmotic pressure. The derivation of $U_{P}$ has been done before by our group.^171^ adapting the work of Halperin^178^ and Zhulina.^179^ $U_{P}$ can be written as

$$\beta U_{P}=\left\{\begin{array}{ll} V_{R}{\sigma_{P}}^{-\frac{3}{2}}{\left(1-\delta_{P}^{2}\right)}^{\frac{9}{4}} & \text{for}\;\delta_{P}\;\varepsilon \;[0:1]\\ 0, & \text{otherwise} \end{array}\right.,$$ (10)

where $V_{R}$ is the volume of the receptor tip, and $\sigma_{P}$ is the area per polymer chain. It is important to note that $\sigma_{P}$ changes along the brush height, $h_{P}$, as a function of the NP radius due to the curvature of the NP. Bigger NPs have a denser polymer brush where single polymer chains are densely packed occupying a smaller $\sigma_{P}$, resulting in higher $U_{P}$. Full derivation can be found in Ref. 174. Equation. (10) explains the effect of the ligand accessibility, given by the inference parameter $\delta_{P}\;\varepsilon \;[0:1]$. $\delta_{P}$ serves as a modulator for the magnitude of $U_{P}$. It signifies the relative length of the ligand tether in comparison to the NP brush. For $\delta_{P}=1$, the ligands are unprotected on the NP surface negating the effect of $U_{P}$. As a result, Eq. (9) accounts for the unspecific interaction between the polymeric chains and the receptors affecting the effective binding energy, ${\Delta G}_{i}^{0}$.

A second source of repulsion originates at the level of the sugar-rich cushion covering the cells, namely, the cell glycocalyx. The latter precedes the $U_{P}$ felt by the receptors, as the glycocalyx repulsion $U_{\mathrm{GAG}}$ affects the bulk NP when it is immersing in the glycocalyx and compressing the sugar chains. Using the same model for $U_{P}$, $U_{\mathrm{GAG}}$ can be derived. The elastic component can be neglected as NP optimal radius ranges from 20 to 40 nm^180^ and GAG chains are long as 100 nm,^181,182^ being NP smaller than GAG height ${R<h}_{\mathrm{GAG}}$. Considering only the osmotic pressure from excluded volumes, $U_{\mathrm{GAG}}$ can be written as

$$\beta U_{\mathrm{GAG}}=\left\{\begin{array}{ll} V_{NP}{\sigma_{\mathrm{GAG}}}^{-\frac{3}{2}}{\left(1-\delta_{\mathrm{GAG}}^{2}\right)}^{\frac{9}{4}} & \text{for}\;\delta_{\mathrm{GAG}}\;\varepsilon \;[0:1]\\ 0, & \text{otherwise}, \end{array}\right.$$ (11)

where $V_{NP}$ is the volume of the NP. $\sigma_{\mathrm{GAG}}$ is the area per GAG chain and can also be expressed in terms of glycans density on the cell membrane (experimentally measured).^173^ The inference parameter $\delta_{\mathrm{GAG}}\;\varepsilon \;[0:1]$ relates the relative height of the receptors, $h_{R}$, with the GAG chains $\delta_{\mathrm{GAG}}=h_{\mathrm{GAG}}/h_{R}$. We can now write the total partition function of a NP considering the attractive and the repulsive contributions as

$$Q=\left[\sum_{i=1}^{\text{min}\left(N_{L},N_{R}\right)}\;\Omega \left(i\right)e^{-i\beta {(\Delta G}_{i}^{0}+U_{P})}\right]e^{-i\beta U_{\mathrm{GAG}}}.$$ (12)

Box 4. Effective binding energy

The effective binding energy or the total free energy $E_{\mathrm{Tot}}$ can be derived from the canonical partition function as −βE=ln(Q), as follows:

$$-\beta E_{\mathrm{Tot}}=\ln\left[\sum_{i=1}^{\text{min}\left(N_{L},N_{R}\right)}\;\Omega \left(i\right)e^{-i\beta {(\Delta G}_{i}^{0}+U_{P})}\right]+U_{\mathrm{GAG}}.$$ (13)

Equation (13) is relatively easy to solve through modeling by introducing the geometrical parameters of the system such as NP size given by its radius R, the height of the binding species, and the steric polymers, along with the ligand–receptor bond energy, and the density of receptors (entry receptors and glycans) on the targeted cell.

Multivalency is the design principle that converts low-affinity L–R interactions into multivalent high-avidity associations. Such enhancement of the strength of the interaction is mostly due to a decrease in the rate of dissociation (*k*_off_) of the multivalent entity (Box 5).^145^ The decisive factor here is how multiple binding modules, including natural and synthetic ligands, are assembled into suitable scaffolds and architectures rather than the supramolecular interactions of individual binding modules. Combinatorial effects lead to a superselective response of the multivalent structure to small changes in the system, such as the number of cell surface receptors.^160^ Eventually, over a critical receptor (or ligand) number threshold the system displays a nonlinear response switching from minimum surface coverage to very high surface coverage.^134^ This superselective response can be accompanied by a range selectivity that defines an upper limit above which repulsive contributions dominate, and NP binding turns null, observing a non-monotonic behavior within a specific region of receptor density.^172^ Ultimately, upgrading the selectivity.

Box 5. Chemical reaction representation of statistical mechanics

The bond energy of the microscopic associations that define the partition function of the system can be decomposed into the experimentally measurable parameter $K_{A}$, known as the association constant. $K_{A}=\frac{k_{\mathrm{on}}}{k_{\text{off}}}$, defines the ligand affinity, where $k_{\mathrm{on}}$ and $k_{\text{off}}$ are the binding and unbinding rates of the free ligands and receptors in solution. The free energy of binding ${\Delta g}_{i}^{0}$ is related to the affinity as^160,168,171^

$$K_{A}=\frac{e^{-\beta {\Delta g}_{i}^{0}}}{\rho_{0}},$$ (14)

where $\rho_{0}=1M$ is the standard concentration of ligands and receptors at which the equilibrium constant is measured.^171^ Moreover, the equilibrium constant can be calculated by experimentally measuring the molar concentrations of bound [LR] ligand–receptor pairs and unbound species [L] and [R] at equilibrium by $K_{A}=\frac{[LR]\;}{\left[L][R\right]}$.

In a multivalent system, the cumulative strength of multiple affinities is denoted by the avidity association constant $K_{av}$, which should be distinguished from the affinity equilibrium constant $K_{A}$ that characterizes the chemical equilibria between a ligand and its receptor. Indeed, $K_{av}$ depends on the individual ligand–receptor affinity $K_{A}$, but also on the valency of the multivalent system $N_{L}$ and on the $N_{R}$. $K_{av}$ includes all possible bound states and is related to $E_{\mathrm{Tot}}$ which measures the overall strength of the multivalent interaction by Dubacheva *et al.*^168^

$$K_{av}=\frac{e^{-\beta E_{\mathrm{Tot}}}}{\rho_{0}}.$$ (15)

The theoretical model presented in this article has been experimentally validated in previous works of our group. Such studies include the implementation of multivalent polymersomes (i.e., self-assembled polymer-based-NP) functionalized with Angiopep-2 (AP2) ligands specific for the LRP1 receptor to target brain endothelial cells due to their important role in the crossing of the blood-brain barrier (BBB).^171,172^ Other works exploit the promiscuous interaction of the synthetic polymeric ligand poly(2-(methacryloyloxy)ethyl phosphorylcholine) (PMPC) to target myeloid cells,^173^ relevant for anti-inflammatory therapies^183^ and antibacterial strategies.^184^ The use of multivalent particles coated with a single type of ligand has proven to be very effective in the phenotypic targeting. Moreover, the use of a multiplexed-multivalent strategy can foster the selectivity of the carrier toward a particular cell population by making the binding more synergistic. For example, PMPC/AP2 multiplexed polymersomes prove to expand the selectivity range to lower numbers of ligands that alone will not correspond to any interactions.^171^ The group of Frenkel has provided analytical validation of the multicomponent targeting via coarse-grained simulations.^135^ Their system is composed of two different ligand types on a NP and two cognate receptors types on a large (flat) surface. Ligands and receptors are expressed at a specific concentration on each component. We find particularly relevant two main design principles they reveal from the analytical calculations. First, the profile of the NP (in terms of ligand density) should match the density composition of the targeted cell. Second, increasing the distinct L–R combinations enhances the selectivity.^135^ This goes in agreement with the increased selectivity reported for the multiplexed PMPC/AP2 system for brain endothelial cells by shifting the binding to lower NP valency.^171^ The multiplexed targeting would allow for the exploitation of the full information about the cell membrane receptor composition and then design particles that target this specific cell phenotype (Box 6).

Box 6. Multiplexed targeting

The model is adaptable to encompass various receptor/ligand (L–R) combinations, allowing the expression of the binding free energy of nanoparticles (NPs) functionalized with different ligand types targeting distinct receptors ${(R}_{1},\;R_{2},\;\ldots ,\;R_{\zeta }$). This leads to the formation of ζ possible L–R combinations, expressed as

$$H=\sum_{R_{1}{,R}_{2}\ldots {,R}_{\zeta }}E_{\zeta }+E_{2}{+E}_{3}+\cdots +E_{\zeta }\approx \sum_{R_{1}{,R}_{2}\ldots {,R}_{\zeta }}E_{\zeta }+{\left\langle E\right\rangle }_{MB}.$$ (16)

The Hamiltonian, denoted as H a mathematical operator, embodies the total energy of the system. Equation (16) is a multicomponent equation, comprising the sum of energies, $E_{\zeta }$, from various L–R combinations, calculated as per Eq. (13) in the Effective Binding Energy section. Additionally, it incorporates energies arising from interactions among receptors trapped within the multivalent unit, involving two, three, up to ζ body components ( $E_{2,\;}E_{3,\;}{\ldots E}_{\zeta })$. Here, $E_{2}$ represents the energies arising from the interaction of receptor 1, $R_{1}$ and receptor 2, $R_{2}$ ${(R_{1}-R}_{2})$. Subsequently, $E_{3}$ represents the energies from interactions involving ${(R_{1}-R}_{2}),\;{(R_{2}-R}_{3})$, and ${(R_{1}-R}_{3})$ extending up to ζ L–R combinations. In scenarios of increased complexity, applying mean field approximations combines many-body components into a singular mean element, expressed by the second term on the right side of Eq. (16). Depending on whether these components are negative or positive, antagonistic and synergistic scenarios arise from receptor interactions.

Cell phenotype is an indication of the state of health of a tissue. In response to environmental stimulus, cells modify the composition profile of molecules that are exposed on their outer surface. In pathological conditions such as cancer or infection, the glycosylation patterns are altered as well as receptor expression.^185–187^ In this context, it represents a clear advantage of a drug-delivery system that discriminates over cell populations expressing a particular receptor combination and selectively targets disease cells only, significantly reducing undesired effects. Such superselective targeting can be achieved by fine-tuned multivalent NPs functionalized with the right combination of ligands capable of binding to one and only one cell type expressing a unique receptor profile, a phenomenon that we dub “phenotypic targeting.”^173^ Overall, exploiting the full information of the cell phenotype that tells us about the concentration of various receptors on the cell surface (glycans and entry-receptors), engineered NPs can be designed according to target that specific receptor profile.^135,173^ This approach enables the development of highly selective drug-delivery nanosystems, that could potentially allow for the precise release of therapeutic agents exclusively within cell phenotypes associated with a disease. Consequently, this strategy can lead to lower therapeutic doses and fewer side effects arising from off-target interactions, ultimately enhancing the overall effectiveness of the therapy.

#### 3. Superselective NPs against intracellular pathogens

Host-adopted pathogens have developed the ability to divert host response, conquering and establishing a growth-permissive niche within cells. Currently, long-term and high-dose antibiotics remain the main strategy for the clinical treatment of intracellular bacterial infections. However, this approach is limited, in part, by the fact that antibiotics have difficulty penetrating the cell membrane to reach the intracellular bacteria.^188^ Poor membrane permeability of drugs significantly reduces the efficacy of antibiotics and hinders the killing of intracellular pathogens. Additionally, systemic delivery of antibiotics results in poor accumulation of drugs within the site of infection. These factors contribute to subtherapeutic antibiotic concentrations within host cells, promoting antibiotic resistance development. Antimicrobial-resistant bacteria (AMR) cause persistent infections that are difficult, and sometimes impossible to treat with currently developed antibiotics.^189^ AMR is causing increased patient mortality and medical costs representing a serious global emergency.^190^

One approach to enhance antibiotic effectiveness is by augmenting the local antimicrobial agent concentration within infected cells. This can result in effective therapeutic doses at the site of infection while avoiding systemic exposure that causes serious side effects in patients.^191,192^

NPs represent a promising strategy for delivering antimicrobial adjuncts into cells to increase intracellular concentration and antibacterial activity. Nanocarriers can be designed to achieve targeting specificity and penetration of biological barriers, ultimately reaching the intracellular environment where pathogens reside.^193^ Multivalent lipidic and polymeric nanoparticles are being extensively studied and employed to address the various limitations of drug molecules.^192^ NPs functionalized with ligands that can selectively bind to specific cellular receptors can undergo receptor-mediated endocytosis resulting in the internalization of the L–R complex and subsequent intracellular delivery of the NP and the therapeutic agent. Studies show that encapsulation of membrane-impermeable antibiotics within multivalent NPs improves their intracellular delivery and results in appreciable antibacterial activity.^179,194^ Compared to free drugs, this approach has several advantages, including (i) increased drug biodistribution by preventing opsonization and hepatic clearance, (ii) high molecule stability to physiological-like conditions, (iii) phenotypic targeting avoiding off-target side effects, and (iv) improved drug efficacy with consequent reduction of the effective dose for therapy, potentially contributing to the reduction of antimicrobial drug resistance.

Drug encapsulation improves drug properties in several ways, namely, increasing their solubility and avoiding their rapid clearance contributing to increased drug efficacy. However, if the NPs lack targeting specificity for the infection site, the effect of systemic delivery with the associated off-targeted effects persists, hence uncovering the full potential of the delivery systems. Most intracellular bacterial pathogens target macrophages (and epithelial cells) for entry, mainly due to their tissue localization and macrophage scavenging activity.^188^ Based on this, macrophages represent a key target for drug delivery against many intracellular pathogens.

Macrophage targeting strategies include functionalizing different ligands on the NP surface, such as antibodies, peptides, proteins, and carbohydrates, to target macrophage-expressed receptors.^195^ Most of the studies exploit the targeting of the CD206 mannose receptor with mannosylated carriers.^195–197^ Also, folate receptors (FRs) have been exploited for targeting with folic acid (essential endogenous vitamin) decorated NPs.^198,199^ FR is overexpressed in activated macrophages in inflammatory conditions.^200^ Also, hyaluronic acid (HA) receptors like the CD44, which is overexpressed in inflammatory conditions, can be targeted with HA-tagged nanosuspensions.^201,202^

Such delivery strategies do great at targeting macrophages in the infection site but there needs to be evidence of the capability to discriminate between infected and healthy macrophages. This is likely because the targeted receptors are common-bearing in healthy and infected cells. Such discrimination demands sophisticated nanomedicines that, sustained by the theory presented previously, require multivalent-multiplexed associations for the discrimination of receptors density profiles on the target. This requires selecting a combination of receptors (more than one type) expressed by the targeted cell population and designing NPs functionalized with a library of reciprocal ligands. It is most probable that a single receptor type is similarly expressed rather than two (or more) receptor types to be equally present in numbers on the targeted cell. Hence, an infected or healthy macrophage will be discriminated by the combination of receptors that uniquely identify that cell phenotype. In such a manner, nanomedicine with active targeting strategies holds great potential in directing drug molecules to their intended sites, thus advancing our efforts to eliminate harmful intracellular pathogens.^184^

## IV. CONCLUSIONS AND FUTURE PERSPECTIVES

Given the heterogeneity of tissue macrophages and the dynamic phenotypes they adopt in inflammatory conditions, macrophage-targeting nanomedicines represent a revolutionary system for the treatment of virtually all major human diseases including infection, chronic inflammatory diseases (such as rheumatoid arthritis, fibrosis, and atherosclerosis), neurodegenerative diseases, and cancer.^28,173,183,195^ However, a major challenge lies in accurately identifying markers for disease-associated macrophages. To overcome this challenge, a comprehensive understanding of macrophage biology is required, including the characterization of macrophage subsets considering their origin and their interdependent relationship with macrophage function. The perfect complexity of nature makes biological systems unique in a way that cell receptors combine forming distinct profiles through which cells exert their function. Identifying these receptor profiles makes it possible to design complementary multivalent units based on multiple bonds to target specific biological targets selectively. Traditionally, it has been believed that the higher ligand affinity (i.e., the most negative binding energy) to its cognate receptor, the higher its ability to target cells or tissues expressing the same receptor. However, such a maximal selectivity at the single molecules imposes that high-affinity ligands results in indiscriminate targeting to any cell expressing the given receptor. In the last decade, the theory of the superselectivity is gradually displacing the current dogma toward multivalent systems based on low-affinity ligands. These superselective systems rely on the collective effect of individual affinities, allowing associations only when receptors are expressed in a specific density, thereby targeting cells expressing the desired receptor while avoiding indiscriminate targeting. We have reviewed the theoretical principles of such multicomponent targeting and how polymer-based NPs can be engineered for rendering the system superselective to the targeted phenotype. Ultimately, they can be used as carriers to deliver drugs more efficiently or exert specific actions and become drugs, effectively transforming them into therapeutic agents.

## Data Availability

Data sharing is not applicable to this article as no new data were created or analyzed in this study.
